# To remain or leave: Dispersal variation and its genetic consequences in benthic freshwater invertebrates

**DOI:** 10.1002/ece3.5656

**Published:** 2019-10-18

**Authors:** Paolo Ruggeri, Ellen Pasternak, Beth Okamura

**Affiliations:** ^1^ Department of Life Sciences Natural History Museum London UK; ^2^ Laboratory of Integrative Biology of Marine Models Station Biologique de Roscoff CNRS‐Sorbonne University Roscoff France; ^3^ Zoology Department Oxford University Oxford UK

**Keywords:** *Cristatella mucedo*, dispersal ability, *Fredericella sultana*, gene flow, hydrological connectivity, statoblasts

## Abstract

Variation in dispersal capacity may influence population genetic variation and relatedness of freshwater animals thus demonstrating how life‐history traits influence patterns and processes that in turn influence biodiversity. The majority of studies have focused on the consequences of dispersal variation in taxa inhabiting riverine systems whose dendritic nature and upstream/downstream gradients facilitate characterizing populations along networks. We undertook extensive, large‐scale investigations of the impacts of hydrological connectivity on population genetic variation in two freshwater bryozoan species whose dispersive propagules (statoblasts) are either attached to surfaces (*Fredericella sultana*) or are released as buoyant stages (*Cristatella mucedo*) and that live primarily in either lotic (*F. sultana*) or lentic environments (*C. mucedo*). Describing population genetic structure in multiple sites characterized by varying degrees of hydrological connectivity within each of three (or four) UK regions enabled us to test the following hypotheses: (1) genetic diversity and gene flow will be more influenced by hydrological connectivity in populations of *C. mucedo* (because *F. sultana* dispersal stages are retained); (2) populations of *F. sultana* will be characterized by greater genetic divergence than those of *C. mucedo* (reflecting their relative dispersal capacities); and (3) genetic variation will be greatest in *F. sultana* (reflecting a propensity for genetic divergence as a result of its low dispersal potential). We found that hydrological connectivity enhanced genetic diversity and gene flow among *C. mucedo* populations but not in *F. sultana* while higher overall measures of clonal diversity and greater genetic divergence characterized populations of *F. sultana*. We suggest that genetic divergence over time within *F. sultana* populations reflects a general constraint of releasing propagules that might eventually be swept to sea when taxa inhabit running waters. In contrast, taxa that primarily inhabit lakes and ponds may colonize across hydrologically connected regions, establishing genetically related populations. Our study contributes more nuanced views about drivers of population genetic structures in passively dispersing freshwater invertebrates as outlined by the Monopolization Hypothesis (*Acta Oecologica*, 23, 2002, 121) by highlighting how a range of demographic and evolutionary processes reflect life‐history attributes of benthic colonial invertebrates (bryozoans) and cyclically parthenogenetic zooplankton. In addition, growing evidence that genetic divergence may commonly characterize populations of diverse groups of riverine taxa suggests that organisms inhabiting lotic systems may be particularly challenged by environmental change. Such change may predispose riverine populations to extinction as a result of genetic divergence combined with limited dispersal and gene flow.

**Open Research Badges:**

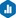

This article has earned an Open Data Badge for making publicly available the digitally‐shareable data necessary to reproduce the reported results. The data is available at https://doi.org/10.5061/dryad.1tm8705.

## Graphical abstract

We undertook extensive, large‐scale investigations of two freshwater bryozoans species whose dispersive propagules (statoblasts) are either attached to surfaces (*Fredericella sultana*) or are released as buoyant stages (*Cristatella mucedo*). Characterizing population genetic structure in multiple sites within each of three (or four) UK regions enabled us to test hypotheses regarding how dispersal variation and hydrological connectivity impact genetic variation in colonial freshwater invertebrates that primarily inhabit lotic (*F. sultana*) or lentic (*C. mucedo*) habitats. We found that hydrological connectivity enhanced genetic diversity and gene flow in and among *C. mucedo* populations but not in *F. sultana*.

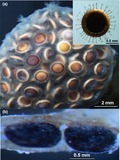

## 1 INTRODUCTION

Freshwater environments are heterogeneous in space and time. Natural drivers of such heterogeneity include flooding, drought, landslides, and the gradual infilling of ponds and lakes with sediments. Additional anthropogenic drivers include canalization, dams, and cultural eutrophication. These various processes produce an array of habitats characterized by varying successional stages as organisms respond to the availability of suitable habitats across the “hydroscape.” For long term persistence, a key feature of aquatic organisms is thus the ability to colonize new sites when their local habitats degrade. Such colonization may confer resilience to local populations by introducing genetic variation that prevents extinction resulting from, for example, genetic drift, population bottlenecks, disease, or environmental change (Allendorf, England, Luikart, Ritchie, & Ryman, 2008; Frankham, 2015). In addition, populations of many organisms are characterized by ongoing metapopulation dynamics whereby extinction in some sites is balanced by colonization of others (Hanski, 1999). Patterns of genetic variation between populations can therefore provide insights into dispersal and metapopulation dynamics, for instance in the form of genetic diversity, gene flow, population differentiation, and bottlenecks (Hanski, 1999; Pannell & Charlesworth, 2000).

Some freshwater residents are capable of active dispersal and can fly, swim, or crawl to colonize appropriate sites. However, animals with limited mobility or that have sessile lifestyles require alternative dispersal mechanisms, such as relying on movements of other animals (zoochory) or drifting in currents (hydrochory) to reach new sites (Bilton, Freeland, & Okamura, 2001). Animal traits that promote variation in dispersal capacity may be expected to vary and thus to influence population genetic variation and relatedness within and among sites (Hughes, Schmidt, & Finn, 2009) and patterns of community composition (Morán‐Ordóñez et al., 2015). Understanding how variation in traits impact dispersal can thus provide insights on patterns and processes that influence freshwater biodiversity. For example, studies on species of mayfly (with winged dispersal stage) and amphipods (dispersal via movement within streams) have revealed greater intrapopulation and overall genetic diversity for mayflies inhabiting streams and reservoirs relative to amphipods (Zickovich & Bohonak, 2007) and decreasing genetic diversity along upstream gradients for populations of an amphipod but not for those of a mayfly (Alp, Keller, Westram, & Robinson, 2012). Comparative analyses of dragonfly and mayfly species with relatively high and low dispersal potentials across groundwater‐fed riverine pool systems in arid environments provided evidence for ongoing gene flow among dragonfly populations and substantial genetic divergence among mayfly populations (Razeng et al., 2017). Finally, populations of mussel species in smaller rivers were characterized by reduced genetic diversity relative to populations of other mussel species in larger rivers—a pattern linked with the relative movements of mussel larvae (glochidia) provided by smaller and larger fish species according to river size (Berg, Christian, & Guttman, 2007).

How variation in traits that impact dispersal may influence population genetic variation and biodiversity in lentic environments has received less investigation. In part this reflects, the low effective dispersal that typifies well‐studied zooplankton taxa and which results from priority effects, local adaptation, and substantial egg banks (De Meester, Gomez, Okamura, & Schwenk, 2002; Mergeay, De Meester, Eggermont, & Verschure, 2011; Okamura & Freeland, 2002). Another likely reason is environmental bias. Upstream/downstream gradients and the dendritic nature of riverine systems enable a straightforward means of characterizing populations and communities along networks (e.g., Hughes et al., 2009; Thomaz, Christie, & Lacey Knowles, 2016). Finally, much of the dispersal‐related research on nonriverine animal taxa has particularly focused on questions concerning the feasibility of dispersal among isolated sites (e.g., Badosa, Frisch, Green, Rico, & Gómez, 2017; Caceres & Soluk, 2002; Louette & De Meester, 2005) and the dispersal mechanisms (e.g., Briski, Bailey, & Mac Isaac, 2011; van Leeuwen, Lovas‐Kiss, Ovegård, & Green, 2017; van Leeuwen & van der Velde, 2012; Sproul et al., 2015) employed by freshwater invertebrates to reach lakes and ponds.

**Figure 1:**
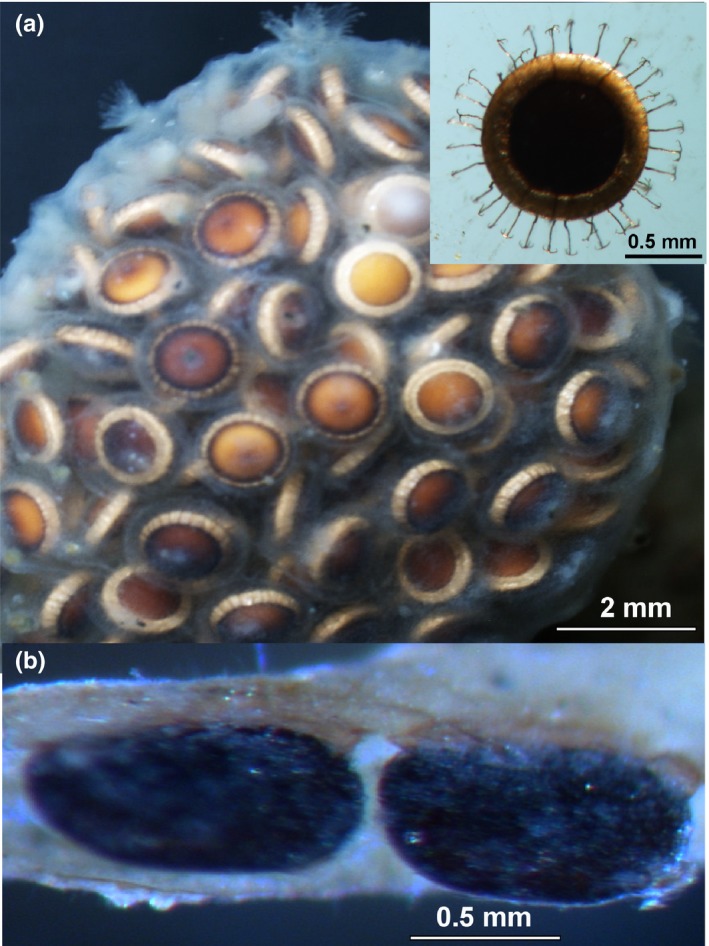
Statoblasts of *Cristatella* and *Fredericella*. (a) Senescing *Cristatella* colony filled with statoblasts enclosed within degrading colony wall at end of growing season (scale bar = 2 mm). Inset shows buoyant, released *Cristatella* statoblast with hooks at tips of radial spines (scale bar = 0.5 mm). (b) Unadorned and nonbuoyant *Fredericella* statoblasts (piptoblasts) retained within persistent colony tube (scale bar = 0.5 mm)

As outlined above, previous studies are biased toward comparing variation in dispersal capacity and its impacts on genetic diversity in populations of taxa in lotic habitats and particularly of insects and fish. More targeted research that addresses dispersal capacity, pathways and consequences for a range of taxa inhabiting lotic and lentic systems should substantially contribute to our general understanding of how aquatic biodiversity is generated and maintained. Accordingly, the aim of this study is to test hypotheses about how differences in dispersal potential and hydrological connectivity will generally influence patterns of genetic variation and divergence in colonial invertebrates that primarily inhabit either lotic or lentic habitats. In particular, we focus on the bryozoan *Cristatella mucedo* that releases buoyant dispersal stages (statoblasts) and lives in still water environments, and the bryozoan *Fredericella sultana* which inhabits flowing water systems and produces statoblasts that remain attached (Figure 1). Previous research has shown that waterbird movements contribute to regular dispersal and gene flow of *C. mucedo*, a process that is likely to be facilitated by spines on statoblasts that attach to feathers. Zoochory may be a particularly important mechanism of dispersal for sessile animals in isolated lakes and ponds. However, buoyant statoblasts may also enable establishment of *C. mucedo* populations across hydrologically connected systems. In contrast, the production of buoyant dispersal stages by residents of lotic systems may ultimately lead to extinction if downstream dispersal greatly exceeds colonization of upstream regions. These issues led us to test the following hypotheses:

1. Patterns of genetic diversity and gene flow will be more influenced by hydrological connectivity in lentic populations of *C. mucedo* (a species with relatively high dispersal potential) than in lotic populations of *F. sultana* (a species with relatively low dispersal potential).
2. As a consequence of its relatively high dispersal capacity, *C. mucedo* will be characterized by less genetic divergence among sites than *F. sultana*.
3. *F. sultana* will demonstrate greater overall genetic variation than *C. mucedo* in keeping with a propensity for genetic divergence linked with its relatively low dispersal potential.

By addressing these hypotheses, our study provides novel evidence regarding the general impact of hydrological connectivity on genetic diversity of invertebrates that vary in dispersal capacity in lotic and lentic environments and the evolutionary consequences of dispersal variation. Our results also provide the first evidence that hydrochory can contribute to the dispersal of freshwater bryozoans. These issues in turn have implications for how rapidly changing freshwater environments may impact the persistence and conservation of freshwater organisms that vary in dispersal capacity. Below we provide further relevant background information on the biology of *C. mucedo* and *F. sultana*.

### 1.1 Study systems

Freshwater bryozoans (Phylum Bryozoa: Class Phylactolaemata) are common residents of both lotic and lentic freshwater environments (Wood & Okamura, 2005). Along with sponges and hydroids, phylactolaemate bryozoans represent a unique and major functional group comprised of benthic colonial invertebrates whose contributions to freshwater ecosystem processes and biodiversity remain relatively poorly investigated. Bryozoan colonies are comprised of modules (zooids) with tentacular crowns (lophophores) that create feeding currents to capture suspended particles. Colonies are found on natural and man‐made surfaces (e.g., tree roots, submerged branches, rocks, pier pilings, plastics, etc.) that are sheltered from sedimentation. All phylactolaemate bryozoans produce small (~1 mm) dormant stages, called statoblasts, that are asexual buds and hence are genetically identical to the parental colony. Following a period of dormancy during inclement conditions, statoblasts “hatch” and the first zooid of a new colony emerges to commence seasonal growth. In temperate regions, the growing season generally extends from late spring through autumn. A brief phase of sexual reproduction is typical in early summer. At least some species also produce nondormant statoblasts that contribute to multiple statoblast‐derived generations during each growing season (Wood & Okamura, 2005).

The freshwater bryozoan, *C. mucedo*, occurs in calm waters and thus is found primarily in lakes and ponds and occasionally in habitats protected from flow in running water systems throughout the Holarctic (Wood & Okamura, 2005). Research on *C. mucedo* populations provides strong evidence for metapopulation dynamics in the form of local extinction and colonization in space (Okamura, 1997) and time (Okamura et al., 2013) and ongoing gene flow among sites (Freeland, Noble, & Okamura, 2000). Accordingly, *C. mucedo* has proved to be a model system for demonstrating dispersal among freshwater habitats (De Meester et al., 2002; Okamura & Freeland, 2002). *C. mucedo* undergoes extensive clonal propagation via fission of the gelatinous, caterpillar‐like colonies, and the production of buoyant statoblasts (“floatoblasts”) in large numbers at the end of the summer growing season (Figure 1a). These statoblasts are often found attached to feathers via the ring of tiny grappling hooks at the tips of spines that develop along the statoblast margins (Figure 1a). At some stage, buoyancy is lost and the statoblasts become associated with sediments unless they have been deposited earlier in flood debris (Hill et al., 2007) or have become entangled in vegetation. An accompanying body of work has demonstrated ongoing gene flow among populations along migratory flyways (Freeland et al., 2000) and evidence for the presence and viability of *C. mucedo* statoblasts following ingestion by waterbirds (e.g., Charalambidou, Santamaria, & Figuerola, 2003; Mouronval, Guillemain, Canny, & Poirier, 2007). In addition, band recovery data from waterbirds explain a significant proportion of variation in both genetic distance and gene flow among *C. mucedo* populations (Figuerola, Green, & Michot, 2005). The presence of statoblasts in fish guts (Mouronval et al., 2007) suggests that movements of fish or piscivorous birds might also effect dispersal (Okamura, Hartikainen, & Trew, 2019). However, buoyancy of statoblasts would also be expected to confer dispersal along hydrologically connected systems. Such dispersal by hydrochory is an issue that has not been directly investigated for any freshwater bryozoan.

The freshwater bryozoan, *F. sultana*, primarily occurs in running water habitats such as rivers, streams, channels, and interconnected lakes. It is widespread in Europe and also occurs in Asia, Australia, and New Zealand but is rare in North America (Wood & Okamura, 2005). *F. sultana* clonally reproduces via fragmentation, when branches of the tubular colonies detach (Fontes, Hartikainen, Taylor, & Okamura, 2017), and the production of nonbuoyant statoblasts (called “piptoblasts”; Figure 1b). Piptoblasts are retained within overwintering colonies or are attached to surfaces in the microhabitat of the parental colony. Research on *F. sultana* has primarily focused on its role as a fish disease reservoir because it acts as host of the causative agent of salmonid proliferative kidney disease (Okamura, Hartikainen, Schmidt‐Posthaus, & Wahli, 2011). There have been no studies explicitly focusing on dispersal and gene flow of *F. sultana* although a recent study incorporated measures of *F. sultana* eDNA distributions to test a spatially explicit fish disease model within a river network setting (catchment; Carraro et al., 2017). Clearly, the widespread distribution of *F. sultana* reflects occasional dispersal that is likely to be mediated by zoochory (animal‐mediated movement) and hydrochory but such dispersal has not been demonstrated. Abd‐Elfattah, El‐Matbouli, and Kumar (2017) showed that a proportion of *F. sultana* statoblasts ingested by carp (but not brown trout) were viable and thus provide some proof‐of‐concept that fish may effect dispersal. However, no records of *F. sultana* statoblasts in wild caught birds or fish were encountered during preparation of a recent review by one of us (Okamura et al., 2019). This suggests that *F. sultana* statoblasts are relatively rarely ingested, perhaps reflecting their adherent nature and lack of spines. It may also be the case that ingested *F. sultana* statoblasts have not been recognized. Nevertheless, if *F. sultana* were commonly encountered in relatively large numbers in vertebrate digestive tracts, they are likely to have been noted in the literature.

## 2 MATERIALS AND METHODS

### 2.1 Sampling sites and bryozoan collection

**Figure 2:**
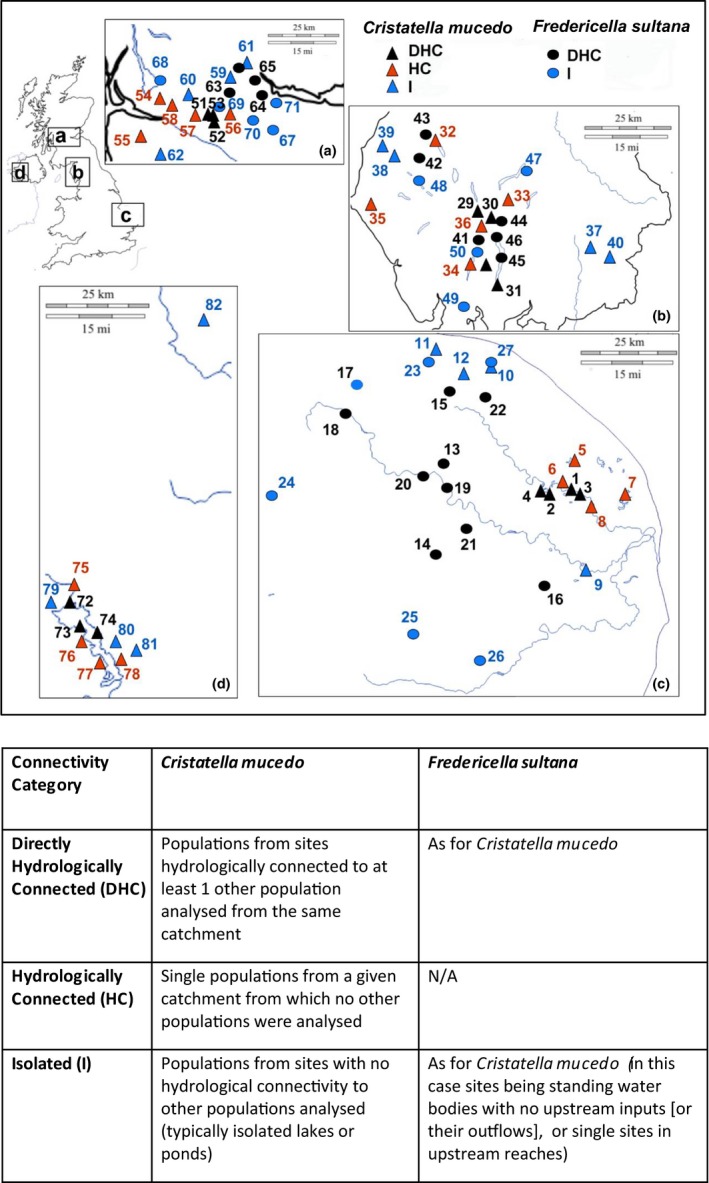
Locations of bryozoan samples subject to genetic analysis across the four UK regions (a = Norfolk, b = Cumbria, c = Greater Glasgow, d = Northern Ireland). *Cristatella mucedo* samples identified by solid triangles (black triangles = Directly Hydrologically Connected sites [DHC]; red triangles = Hydrologically Connected sites [HC]; blue triangles = Isolated [I] sites). *Fredericella sultana* samples identified by solid circles (black circles = Directly Hydrologically Connected [DHC] sites; blue circles = Isolated [I] sites)

To test generality of our results, we collected bryozoans from the following three regions of the UK: Norfolk, Cumbria, Greater Glasgow. The regions can be broadly characterized as lowland, agricultural (Norfolk), upland (Cumbria), and urban (Greater Glasgow; Figure 2). Further collections of *C. mucedo* statoblasts were available from the Lough Erne system in Northern Ireland (another lowland, agricultural landscape). Within each region, we aimed to collect some 15 populations of each species from a series of sites characterized by differences in hydrological connectivity as detailed below. We note that it is impossible to dictate exactly where bryozoan populations will be found as they can be highly patchy over space and time (Okamura, 1997; Okamura et al., 2013). Furthermore, their presence relies on availability of suitable substrata such as wood that has been submerged for a prolonged period, large stones, and tree roots (Fontes, Hartikainen, Williams, & Okamura, 2017; Wood & Okamura, 2005). Our collections therefore could not be restricted to predefined spatial scales but reflected what we managed to sample within each general region and according to hydrological connectivity of sites. In addition, with one exception (*C. mucedo* populations from Northern Ireland), collections were not restricted to populations from the same catchment Populations of the two species were assigned to somewhat different categories of hydrological connectivity in view of differences in dispersal capacities and distributions as described below.

For *C. mucedo*, we adopted three categories of hydrological connectivity. We defined sites as directly hydrologically connected (DHC; Figure 2) when populations were collected from >1 site within a hydrologically connected system. Populations from such sites (*n* = 4 in Norfolk and Cumbria; *n* = 3 in Greater Glasgow; Table S1) were collected from lakes or lochs that were connected by rivers or streams. Single populations collected from sites in different hydrologically connected systems (i.e., similar arrays of connected sites but in different catchments) we simply refer to as hydrologically connected populations (HC; Figure 2; *n* = 5 in Cumbria and Greater Glasgow; *n* = 4 in Norfolk; Table S1). We adopted these categories because populations from HC sites might be expected to have genetic diversity similar to that observed for DHC sites (due to a similar potential for colonization from other populations in the watershed) but may show less evidence of genetic relatedness and gene flow because they are on independent catchments. Single populations were also collected from sites (lakes, ponds) that had no hydrological connections to other water bodies (I; Figure 2; *n* = 4 in each region; Table S1). Sites from which *C. mucedo* populations were collected in Northern Ireland were assigned to roughly similar categories that were reflective of sampling within one large catchment system here. In this case, populations collected from sites in the large Upper Lough Erne were considered to be directly hydrologically connected (DHC; *n* = 3). Single populations collected from satellite lakes with hydrological connection to Upper Lough Erne (via channels and a further satellite lake) were considered to be hydrologically connected (HC; *n* = 4). Isolated sites had no apparent hydrological connections to other water bodies (I; *n* = 4). We note that DHC and HC categories as used here are not entirely consistent with those used for populations in other regions (where more than one catchment was sampled). However, relative trends in connectivity are expected to be similar with greater connectivity of populations within the Upper Lough Erne and a lower degree of connectivity with satellite lakes connected by channels to the Upper Lough Erne.

In the case of *F. sultana*, populations were collected from sites assigned to two categories of hydrological connectivity: directly hydrologically connected (DHC) and isolated (I) (Figure 2). Just as for *C. mucedo*, DHC sites included cases where populations were sampled from >1 site within catchments. Typically, such populations would be collected from two or more sites along a river (including tributaries of that river) or interconnected lake systems. In this case, we included data for populations from different catchments (Figure 2; Norfolk: River Wensum system [*n* = 4]; River Yare system [*n* = 3]; River Bure [*n* = 2]; Cumbria: River Rothay/Brathay [*n* = 4]; River Cocker/Derwent [*n* = 2]; Greater Glasgow: River Carron [*n* = 2]; Forth Clyde Canal [*n* = 2]; Table S1). Isolated sites comprised cases where a single population was collected from isolated sites from unique catchments (*n* = 6 for Norfolk and *n* = 4 for Cumbria and *n* = 5 for Greater Glasgow; Table S1). Such isolated sites included lakes, ponds, and reservoirs with no hydrological connectivity to other upstream sites, outflows from such standing water bodies, and single localities relatively near headwaters of rivers or their tributaries (Table S1). Because *F. sultana* is primarily found in running waters, the array of “isolated sites” as defined here is not equivalent to that of *C. mucedo* (which lacked hydrological connectivity to other water bodies). However, dispersal of *F. sultana* via hydrochory should be much reduced in this array of isolated sites relative to that among the DHC sites.

Mud was collected by wading into shallow regions of water bodies and using a ladle mounted on a pole, or from a boat using an Ekman grab. Statoblasts of *C. mucedo* were collected after passing the mud samples through sieves with mesh sizes of 1.7 mm and 630 μm. The material retained after processing was examined with a stereomicroscope to identify and collect whole and undamaged statoblasts. Statoblasts were then preserved in absolute ethanol and stored at −20°C until DNA extraction. Branches of *F. sultana* were collected from independent colonies growing variously on hard surfaces (e.g., submerged roots of alder trees, submerged branches, rocks, spillways from canals, etc.) and were placed in individual 50‐ml falcon tubes filled with water from the collecting site. Following their collection, the branches (henceforth referred to as colonies) were observed with a stereomicroscope to confirm colonies were alive (the presence of digestive tracts, lophophores) and were then preserved in absolute ethanol and stored at −20°C until DNA extraction.

Our general sampling strategy was to collect some 30 samples per site for multilocus genotyping of each bryozoan species.

### 2.2 DNA extraction and microsatellite genotyping

Genomic DNA (gDNA) for both species was isolated using the HotShot protocol (Truett et al., 2000) specifically designed for small amount of tissues (method described in Appendix S1). Samples were screened at 10 microsatellite loci per species (Table S3). Details of PCR and profiling are provided in Appendix S1.

Fragment analysis was carried on an ABI 3730 DNA Analyzer (Applied Biosystems), and genotype peaks were scored using Peakscanner 2.0 (Applied Biosystems). Allele calling was performed with FlexiBin v2 (Amos et al., 2006). Each genotypic dataset was checked for quality in terms of (a) missing data, (b) repeatability, and (c) scoring errors (stuttering bands, allele dropouts, and null alleles). Repeatability was checked by re‐calling alleles for a subset of randomly chosen samples (~10% per dataset) and comparing them with previous scoring. The possibility for scoring errors was tested via Microchecker 2.2.3 (van Oosterhout, Hutchinson, Wills, & Shipley, 2004) and data corrected for null alleles using the Brookfield algorithm (Brookfield, 1996).

### 2.3 Estimates of genotypic and genetic diversity

The software GenClone2.0 (Arnaud‐Haond & Belkhir, 2007) was used to detect multilocus genotypes (hereafter MLGs) and to obtain clonal diversity estimates. Individuals matching for the same multilocus profile were considered products of asexual reproduction and treated as identical MLGs. In keeping with standard approaches for population genetic analyses of clonal organisms (e.g., Combosch & Vollmer, 2011; Hamorova, Mergeay, & Petrusek, 2011), only unique MLGs were retained for subsequent analysis of each population to avoid redundancy and bias in the estimation of population genetic parameters. Clonal diversity was estimated as: (a) *total clonal diversity* (total number of unique MLGs), (b) *population clonal diversity* (*N*_g_; the number of unique MLGs in a population), and (c) a *genotypic richness index* (*R*; *R* = *N*_g_−1/*N*−1 where *N* is the number of individuals; a standardized measure of population genotypic diversity that accounts for sample size; Dorken & Eckert, 2001).

The mean number of alleles (*N*_A_), allelic richness (*R*_S_), observed (*H*_O_), expected heterozygosity (*H*_E_), inbreeding coefficient (*F*_IS_), population differentiation (*F*_ST_), and deviation from Hardy–Weinberg equilibrium (HWE) were calculated using FSTAT 2.9.4 (Goudet, 2001). Pairwise tests for assessing linkage disequilibrium among loci were carried out using the online version of Genepop 4.6 (Rousset, 2008; http://genepop.curtin.edu.au/).

### 2.4 Relating genetic diversity to hydrological connectivity

Several approaches were used to determine whether genetic diversity within sites is influenced by hydrological connectivity. We determined if identical clones occurred in multiple sites by comparing MLGs obtained from GenClone2.0 (Arnaud‐Haond & Belkhir, 2007). We analyzed whether population differentiation might be influenced by hydrological connectivity by analyses of *F*_ST_ (Weir & Cockerham, 1984; see Table S1). We also characterized present‐day and historical migration rates (evaluated in GENECLASS 2.0 and Migrate‐n v.3.5.1, respectively) and analyzed these rates according to hydrological connectivity of populations. The present‐day level of gene flow was estimated using a Bayesian assignment test implemented in GENECLASS 2.0 (Piry et al., 2004; Rannala & Mountain, 1997). The most probable site of origin of each MLG per population was assessed based on a set of 1,000 Monte Carlo Markov Chain (MCMC) iterations. A *p*‐value of <.01 was used to assess significance. The proportion of MLGs unassigned to the same sample was used as a proxy of immigration (0 to 1 metric with 0 equal to no immigration and 1 equal to all MLGs derived from another site) and hence as a rate of gene flow. Migrate‐*n* (Beerli, 2006; Beerli et al., 2009) uses Bayesian and coalescent statistics to calculate a demographic parameter Θ (Θ = 4*N*_e_ *μ*, where *N*_e_ is the effective population size and *μ* is the mutation rate) and the historical migration rate, *M* (*M* = *m*/*μ*, where *m* is the immigration rate per generation, and *μ* is the mutation rate). A Brownian motion model was used, and mutation was considered constant over the set of 10 microsatellite markers per species. The MCMC procedure consisted of one long chain with 500,000 recorded genealogies for each locus, with 10,000 genealogies discarded as burn‐in.

We used the software PAST (Hammer, Harper, & Ryan, 2001) to perform one‐way ANOVA in order to analyze the relationships of various parameters and hydrological connectivity when data were normally distributed (sometimes requiring logit, mean subtraction, or Box‐Cox transformations). When normal distributions could not be achieved, a nonparametric Kruskal–Wallis test was used.

Finally, we performed principle components analysis (PCA) on samples within landscapes to examine a potential role of hydrological connectivity in segregating sites. We then employed a discriminant analysis of principle components (DAPC; Jombart & Ahmed, 2011; Jombart, Devillard, & Balloux, 2010) to describe the general population structure of both species that accounts for the genetic differences among groups (according to hydrological connectivity) while minimizing the differences within groups using data from principal components analysis (PCA) of genetic variation. We note that DAPC addresses the issue of whether populations in hydrologically connected sites are more genetically related than those from isolated sites and is thus complementary to our other analyses relating to this issue such number of shared MLGs.

### 2.5 Characterizing spatial patterns of genetic diversity

To further infer whether impacts of hydrological connectivity generally influence bryozoan populations, we explored whether bryozoan populations from different regions are genetically distinct. We used similar approaches to those described in the previous section to determine whether measures of genetic diversity vary across regions.

In addition, we determined whether population structure would resolve our data according to their geographical regions using both multivariate and Bayesian methods. The multivariate method implemented in DAPC (Jombart & Ahmed, 2011; Jombart et al., 2010) was used to describe the general population structure of both species. The Bayesian analysis was performed with STRUCTURE version 2.3.4 (Pritchard, Stephens, & Donnelly, 2000). An admixture model and correlated allele frequencies were assumed. Simulations were based on 25,000 discarded iterations (burn‐in) and 250,000 MCMC retained replicates; population structures comprised of between 1 and 48 clusters (*K*) were simulated for *C. mucedo* and between 1 and 34 clusters (*K*) for *F. sultana*. Each *K* was tested five times. The online software STRUCTURE HARVESTER 0.6.92 (Earl & vonHoldt, 2011) was used to evaluate results under the Evanno method expectations (Evanno, Regnaut, & Goudet, 2005) while CLUMPAK server (http://clumpak.tau.ac.il; Kopelman, Mayzel, Jakobsson, Rosenberg, & Mayrose, 2015) was used to obtain graphical plots.

### 2.6 Evidence for genetic divergence, selection, and bottlenecks

We employed Mantel tests (using Genepop (ISOLDE) [Rousset, 2008]; http://genepop.curtin.edu.au/) to gain evidence for genetic divergence by determining whether *F*_ST_ values were correlated with geographical distance among sampling sites (according to Isolation by Distance theory). For each dataset, a semi‐matrix of pairwise genetic distances (*F*_ST_) between samples was created and regressed against the corresponding shortest linear distances between samples with a permutation test of 10,000 iterations.

Neutrality of markers was explored using (a) an *F*_ST_‐based test was carried out in Arlequin (Excoffier, Hofer, & Foll, 2009; Excoffier & Lischer, 2010) and (b) a Bayesian method developed in BayeScan2.1 (Foll & Gaggiotti, 2008).The *F*_ST_‐based test involved 20,000 simulations (each composed of 100 demes per group and 10 groups) and was repeated under various sets of combinations to examine associations with hydrological connectivity and regions. A preliminary test was conducted to identify outlier loci using the whole dataset. Pairwise tests were performed between samples collected from different “populations” as revealed by DAPC and STRUCTURE analyses (Norfolk, Cumbria, Greater Glasgow/Northern Ireland; Table 1) and between samples collected from different hydrological connectivity regimes within each “population” (Table 1). A Bonferroni correction method was applied to increase the level of accuracy in avoiding false‐positive tests. The Bayesian approach to test marker neutrality used BayeScan2.1 with a default setting (20 independent runs with 5,000 iterations each, 50,000 iterations burn‐in, sample sizes of 5,000 and thinning of 10). Non‐neutrality in BayeScan2.1 results was inferred following the Jeffrey's interpretation, as suggested in the software User Guide.

**Table 1:** Tests to detect outlier loci. (A) Hierarchical Island Method (HIM) test performed in Arlequin. (B) Test performed with Bayescan2.1

(A)							
*C. mucedo*							
Locus	Overall	Population comparisons connectivity					
		Norfolk vs. Cumbria	GG‐NI vs. Cumbria	Norfolk vs. GG‐NI	Norfolk	Cumbria	GG‐NI
Cmu2.5	0.160	0.144	0.151	0.157	**0.067** †	0.127	0.143
Cmu2.3	0.211	**0.270** †	0.152	0.199	0.216	0.161	0.118
Cmu9.4	0.240	0.267	0.186	0.238	0.223	0.188	0.139
Cmu6.7	0.251	0.207	0.242	0.253	0.106	0.063	**0.283** †
Cmu7.5	**0.262** †	0.251	**0.248** †	**0.238** †	0.181	0.246	0.196
Cmu5.5	0.181	0.187	0.157	0.167	0.155	0.147	0.119
Cmu3.6	0.165	0.167	0.150	0.151	0.110	0.148	0.112
Cmu1.1	0.220	0.218	0.207	0.211	0.130	0.158	0.207
Cmu9.3	NA	NA	NA	NA	NA	NA	NA
Cmu2.2	0.218	0.211	0.229	0.238	0.133	0.221	0.174

*F. sultana*							
Locus	Overall	Population comparisons connectivity					
		Norfolk vs. Cumbria	Glasgow vs. Cumbria	Norfolk vs. Glasgow	Norfolk	Cumbria	Glasgow
Fs09	NA	NA	0.145	NA	NA	0.132	0.123
Fs09TKU	0.149	0.168	0.136	0.176	0.189	0.106	0.112
Fs27	**0.143** **†**	0.173	0.142	0.134	0.137	0.168	**0.052** **†**
Fs14TKU	**0.349** ***	**0.522** ***	**0.367** **†**	**0.268** **†**	0.137	0.100	**0.206** **†**
Fs24TKU	0.215	0.255	0.289	0.118	0.114	0.220	0.105
Fs13	**0.135** **†**	**0.143** **†**	**0.127** **†**	0.180	0.136	**0.081** **†**	0.138
Fs25TKU	0.175	0.196	0.225	0.152	0.111	0.155	0.111
Fs04	**0.260** **†**	0.305	0.273	**0.243** **†**	0.121	**0.291** ***	**0.198** **†**
Fs18	0.225	0.277	0.294	0.134	0.140	0.210	0.164
Fs12TKU	0.187	0.224	0.153	0.215	**0.218** **†**	0.136	0.133

(B)					
*C. mucedo*					
Locus	Prob	log_10_(PO)	*q*val	*F*_ST_	Jeffreys' interpretation
Cmu2.5	0.702	0.371	0.195	0.222	ns
Cmu2.3	0.023	−1.620	0.658	0.271	ns
Cmu9.4	0.019	−1.708	0.765	0.272	ns
Cmu6.7	0.043	−1.343	0.594	0.273	ns
Cmu7.5	0.023	−1.632	0.703	0.272	ns
Cmu5.5	0.330	−0.307	0.354	0.250	ns
**Cmu3.6**	**0.908**	**0.992**	**0.092**	**0.204**	**Substancial**
Cmu1.1	0.016	−1.789	0.787	0.272	ns
Cmu9.3	0.020	−1.695	0.738	0.271	ns
Cmu2.2	0.048	−1.297	0.503	0.274	ns
*F. sultana*					
Fs09	0.067	−1.141	0.472	0.236	ns
**Fs09TKU**	**0.982**	**1.737**	**0.018**	**0.169**	**Very strong**
Fs27	0.121	−0.859	0.395	0.231	ns
**Fs14TKU**	**0.881**	**0.871**	**0.068**	**0.335**	**Substancial**
Fs24TKU	0.033	−1.461	0.581	0.241	ns
**Fs13**	**0.844**	**0.734**	**0.097**	**0.186**	**Substancial**
Fs25TKU	0.131	−0.823	0.298	0.232	ns
Fs4	0.043	−1.343	0.532	0.241	ns
Fs18	0.025	−1.591	0.620	0.240	ns
Fs12TKU	0.670	0.307	0.156	0.199	ns

Note

Bold indicates significant values at *p* < .05.

Prob = probability of loci significant (if larger than 0.76) for non‐neutral expectation. Log_10_(PO) = Logarithm (in base‐10) of the Posterior Odd (PO) probability (must be larger than 0.5 to be significant); *Q*val = the *q*‐value of given locus which is the minimum FDR (false discovery rate) at which each locus may become significant; *F*_ST_ = genetic differentiation index; Jeffreys' interpretation results listed as ns = nonsignificant, substancial (*p* < .05), strong (*p* < .01), very strong and decisive (*p* < .001).

^†^ Loci significant (*p* < .05) for the original test but nonsignificant after the application of the Bonferroni correction.

*** Loci significant after the Bonferroni correction.

We tested for genetic signatures derived from recent population bottlenecks using a method implemented in BOTTLENECK 1.2 (Piry, Luikart, & Cornuet, 1999). This test is based on a “heterozygosity excess theory” that assumes how the number of alleles declines faster than expected heterozygosity (*H*_E_) during a strong event of reduction in population size. The expected heterozygosity at mutation–drift equilibrium (*H*_eq_) derives from the number of observed alleles. Hence, a rapid decline in alleles results in *H*_E_ being larger than *H*_eq_, producing a heterozygosity excess (*H*_exc_; Cornuet & Luikart, 1996). Values of *H*_exc_ were tested by Wilcoxon's signed‐rank test using 1,000 iterations and two distinct mutational models: a stepwise mutation model (SMM) and a two‐phase mutation model (TPM) with 95% single‐step mutations and 5% multistep mutations. An additional test implemented by BOTTLENECK 1.2 based on allele frequency shift (Shift‐Mode test) was used to further substantiate the occurrence of a recent bottleneck. Samples significant for all the three tests were considered as bottlenecked. Samples with <4 MLGs were removed because the sample size was too small for processing by BOTTLENECK 1.2.

## 3 RESULTS

### 3.1 Dataset and genotyping quality

We collected 4,011 statoblasts of *C. mucedo* from sediments. Of these 1,349 statoblasts were successfully genotyped from 48 sites across Norfolk, Cumbria, Greater Glasgow and Northern Ireland (Table S1). In the case of *F. sultana* 1,336 colonies were collected and 993 individuals successfully genotyped from 34 sites in Norfolk, Cumbria, and Greater Glasgow (Table S1).

Missing genotypes accounted for 6.5% of the *F. sultana* dataset and 22.9% of the *C. mucedo* dataset. We re‐genotyped 96 individuals and 138 individuals of *F. sultana* and *C. mucedo*, respectively, and none were dissimilar to the original calling.

Allele dropout and stuttering were consistently absent. Null alleles were detected in 45 of 270 tests for *F. sultana*. After the Brookfield correction, 30 of 270 (11.11%) remained significant. Null alleles were detected in 15 of 330 tests for *C. mucedo* with 2 of 330 (0.6%) remaining significant after the Brookfield correction. Because the Brookfield correction improved both datasets and no loci showed a systematic presence of null alleles, both corrected datasets were used for subsequent statistical analyses.

### 3.2 Overall patterns of genetic diversity

*Cristatella mucedo* and *F. sultana* were characterized by 269 and 337 unique clones, respectively, across all sites and regions. Six of the 34 populations of *F. sultana* were monoclonal while all 48 populations of *C. mucedo* presented >1 MLG (Table S1; *z* test, *Z* = 3.019, *p* < .01). Nevertheless, average clonal diversity (*N*_C_) and average genotype richness (*R*) within sites were significantly greater for *F. sultana* (*N*_C_: 5.88 ± 2.87 *SD* [*n* = 48] and 10.00 ± 6.27 *SD* [*n* = 34] for *C. mucedo* and *F. sultana*, respectively, Kruskal–Wallis test for equal medians; *p* < .01; *R*: 0.18 ± 0.11 *SD* [*n* = 48] and 0.33 ± 0.23 *SD* [*n* = 34] for *C. mucedo* and *F. sultana*, respectively, Kruskal–Wallis test for equal medians; *p* < .01; Table S1).

Patterns of expected heterozygosity (*H*_E_), observed heterozygosity (*H*_O_), number of alleles (*N*_A_), and inbreeding (*F*_IS_) showed relatively similar ranges across populations of both *C. mucedo* and *F. sultana* (Table S1). *F*_IS_ was significantly larger than expected under HWE (Hardy–Weinberg equilibrium) in 3 of 48 *C. mucedo* populations (Esthwaite [EST]; Bardowie Loch [BAR]; Carry Bridge [CAB]) and in 4 of 34 *F. sultana* populations (Bowness marina [BOW]; Windermere [WIM]; Forth Clyde Canal 1 [FCC]; Forth Clyde Canal 2 [FCD]; Table S1). For both species, 3 of the 10 microsatellite loci were not in HWE (Cmu5.5, Cmu1.1, and Cmu9.3 in *C. mucedo*; Fsu09, Fsu09TKU, and Fsu04 in *F. sultana*) and the overall test for HWE incorporating all populations was also significant (*p* < .001), indicating a general lack of conformation to HWE in both species. For *F. sultana*, there was strong evidence for linkage disequilibrium (LD) between loci in two cases (Fs04 and Fs12TKU, Fs13 and Fs12TKU; pairwise tests for linkage disequilibrium, *p* < .001). Tests were carried out excluding linked loci from the dataset in order to evaluate their impact on characterizing clonal and genetic variation. No impact was observed when excluding the linked loci Fs04, Fs12TKU, and Fs13 and therefore, these loci were retained in the final analysis. There was no evidence for linkage disequilibrium among populations of *C. mucedo*.

### 3.3 Genetic diversity and hydrological connectivity

There were 15 cases of *C. mucedo* clones shared variously among directly connected sites (Wroxham Broad [WRO]; Hoveton Broad [HOV]; South Walsham Broad [SWA]; Cockshoot Broad [CKS] in Norfolk [*n* = 8]; Grasmere [GSM]; Rydal Water [RYD]; Windermere [WIN] in Cumbria [*n* = 4]; Lochend [LND]; Bishop Loch [BIS]; Woodend Loch [WDN] in Greater Glasgow [*n* = 3]; Table S2). There was a single case of a clone shared between isolated sites (Ullock Pond [ULP]; Mockerkin Tarn [MOK] in Cumbria). A goodness‐of‐fit test indicated that the distribution of shared *C. mucedo* clones was dependent on hydrological connectivity (*χ* ^2^ test: 160.97, *p* < .001; based on null hypothesis that shared clones are equally likely in isolated, HC and DHC sites). Three cases of shared clones were found for *F. sultana* (Table S2). One instance was between hydrologically isolated sites in different catchments (River Wensum [WEN] and River Stiffkey [STI] in Norfolk). Two cases were between DHC sites (River Carron 1 [RCD] and River Carron 2 [CRN] in Greater Glasgow).

**Figure 3:**
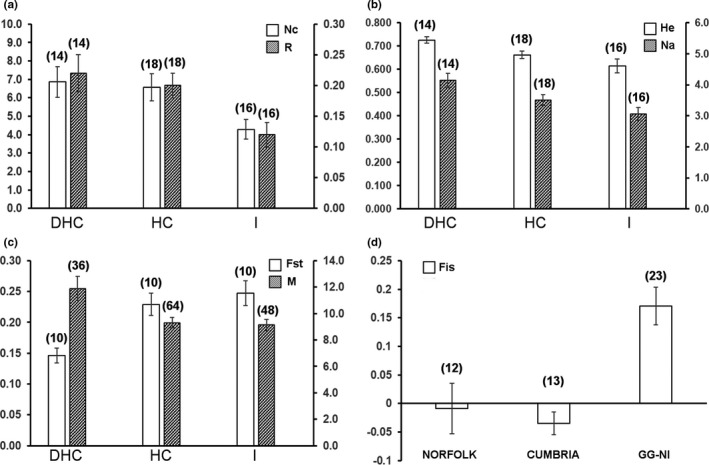
Average values (and *n*‐values) with standard error bars for parameters that significantly varied with hydrological connectivity (a, b, c) and region (d) in *Cristatella mucedo*. (a) *N*_C_ = mean number of clones; *R* = mean genotypic richness; (b) *H*_E_ = expected heterozygosity; *N*_A_ = mean number of alleles; (c) *F*_ST_ = genetic differentiation index; *M* = averaged gene flow estimates (derived from Migrate‐n; Beerli, 2006; Beerli et al., 2009); (d) *F*_IS_ = inbreeding coefficient index

**Figure 4:**
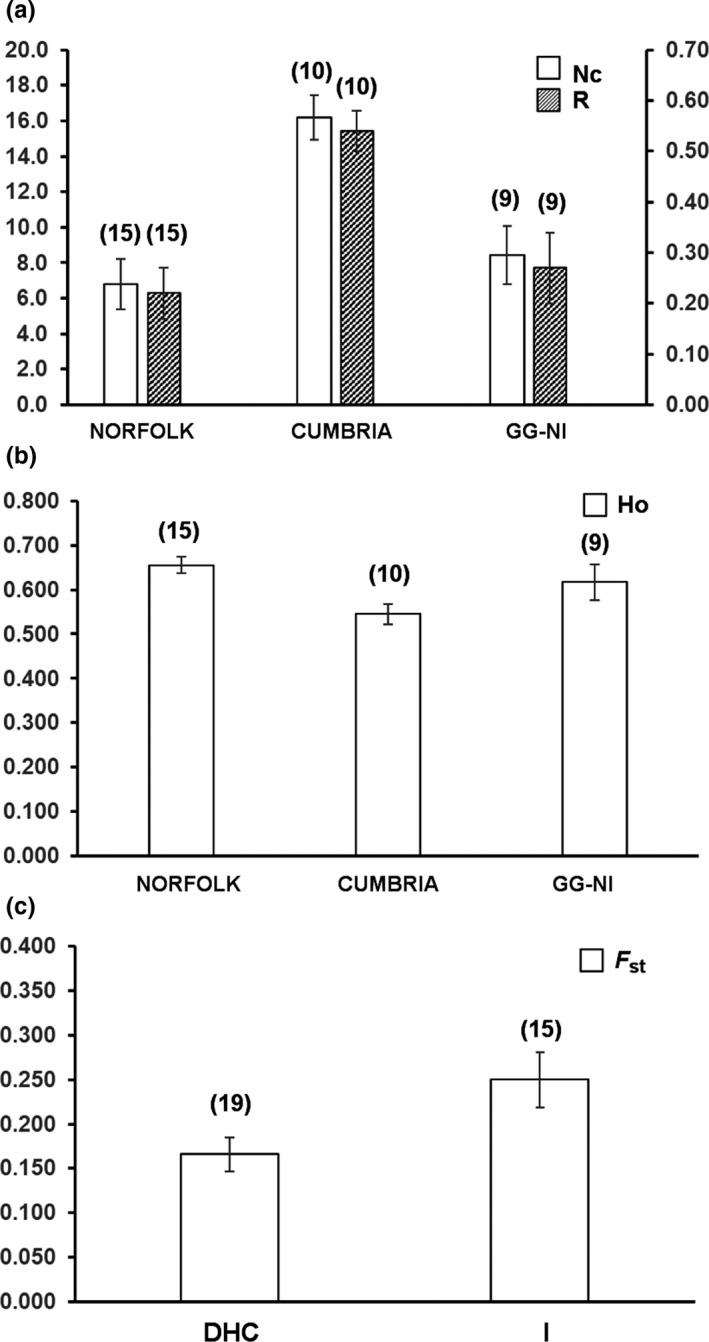
Average values (and *n*‐values) with standard error bars for parameters that significantly varied with region (a, b) and hydrological connectivity (c) in *Fredericella sultana*. *F*_ST_ = genetic differentiation index; *N*_C_ = mean number of clones; *R* = mean genotypic richness; *H*_O_ = observed heterozygosity

Patterns of clonal diversity varied with hydrological connectivity for *C. mucedo* with clonal diversity (*N*_C_: ANOVA, *p* < .05) and richness (*R*: ANOVA, *p* < .05) being reduced in isolated relative to connected sites (DHC and HC; Figure 3, Table S4). Clonal diversity and richness in *F. sultana* were not influenced by hydrological connectivity (*N*_C_: Kruskal–Wallis, *p* > .05; *R*: ANOVA, *p* > .05; Table S4). *H*_E_ and *N*_A_ in *C. mucedo* were significantly smaller in isolated than in directly connected sites (*H*_E_: ANOVA, *p* < .01; *N*_A_: ANOVA, *p* < .01) while *F*_ST_ was significantly larger in HC and isolated sites than in DHC sites (ANOVA, *p* < .001; Figure 3; Table S4). *F*_ST_ for *F. sultana* was larger in isolated than in DHC sites (ANOVA; *p* < .05; Figure 4, Table S4). There was no evidence that hydrological connectivity influenced other estimates of genetic diversity in both *C. mucedo* and *F. sultana* populations (ANOVA tests all with *p* > .05; Table S4). The historical migration rate (*M*) values (derived from Migrate‐n) were significantly (Kruskal–Wallis, *p* < .05) larger in DHC sites than in HC and isolated sites for *C. mucedo* (Figure 3, Table S4). M values did not vary with hydrological connectivity in *F. sultana* (ANOVA, *p* > .05; Table S4). In view of the discrepancy in hydrological connectivity categories used for *C. mucedo* populations collected from the single large catchment in Northern Ireland and those from Norfolk, Cumbria, and Greater Glasgow, we also undertook analysis of data excluding the samples from Northern Ireland. The analysis revealed similar results to those summarized above although support for higher clonal diversity and richness in hydrologically connected sites was not quite significant (*N*_C_: ANOVA, *p* = .100; *R*: ANOVA, *p* = .086; Table S4).

**Figure 5:**
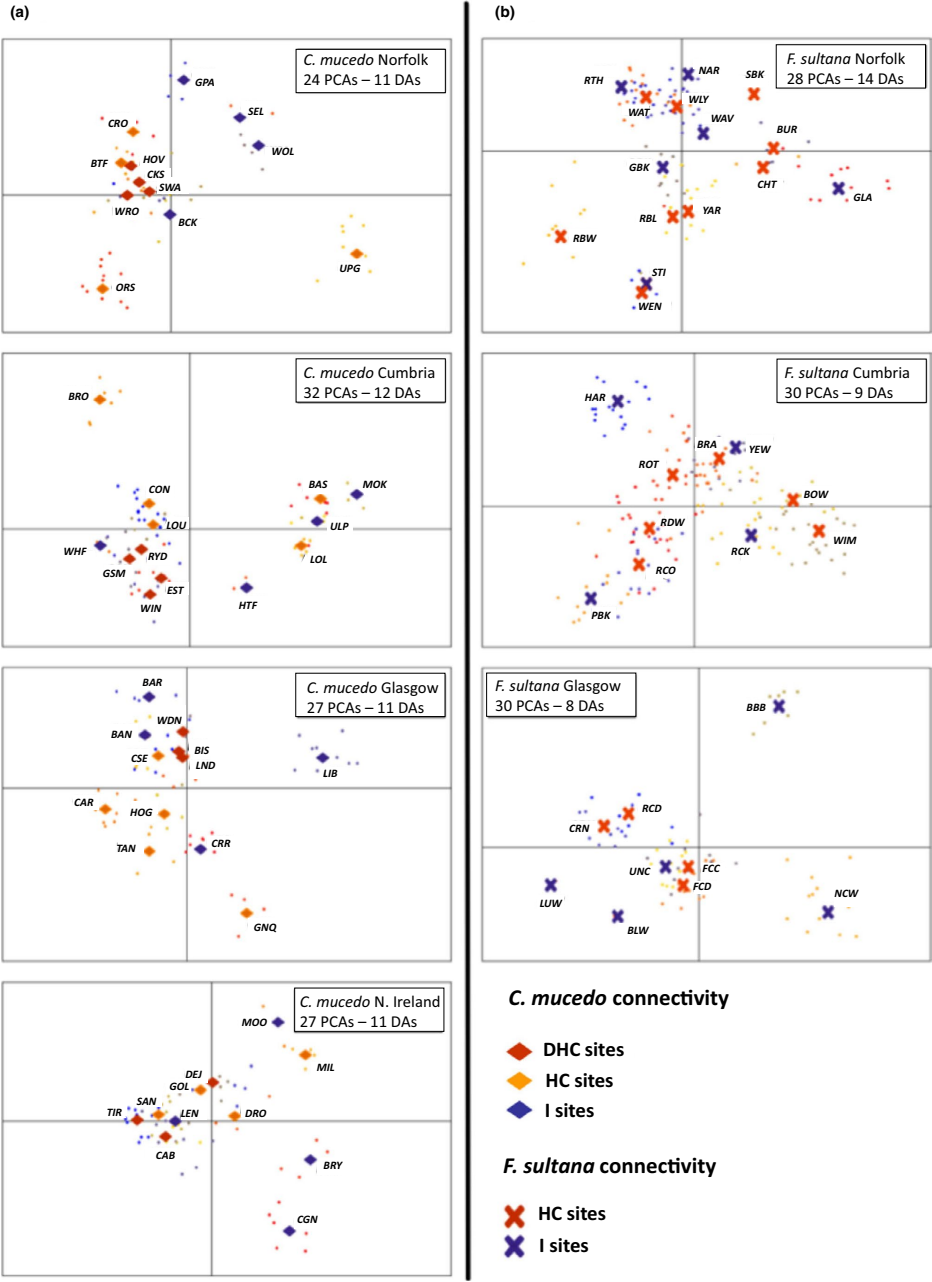
Regional DAPC plots of local population structure in relation to hydrological connectivity for *Cristatella mucedo* (a) and *Fredericella sultana* (b). Plots of genetic relatedness among unique MLGs (small dots) along the first (horizontal) and second (vertical) most significant discriminant axes (DAs). Diamonds and crosses represent, respectively, the centered area of plots for each bryozoan population (identified by population codes; Table S1). Symbol colors signify hydrological connectivity as per key. DHC, Directly Hydrologically Connected; HC, Hydrologically Connected; I, Isolated sites (I) sites. Boxes provide details for region, number of PCAs, and significant discriminant axes (DAs) retained in each plot

Results from the general discriminant analyses of principle components (DAPC) were broadly consistent with the above results. DHC, HC and isolated *C. mucedo* populations were separated by hydrological connectivity along the main DA for Norfolk (variance explained along the main [horizontal] axis = 86.95%), Cumbria (variance explained along the main axis = 92.41%), and Northern Ireland (variance explained along the main axis = 97.14%; Figure 5a). PCA of the *C. mucedo* samples in Glasgow did not provide clear evidence for an effect of hydrological connectivity (variance explained along the main axis = 95.31%; Figure 5a). There was no suggestion that hydrological connectivity separated DHC or isolated *F. sultana* populations from Norfolk (variance explained along the main axis = 99.01%), Cumbria (variance explained along the main axis = 93.82%), or Glasgow (variance explained along the main axis = 97.37%; Figure 5b).

The assignment test with GENECLASS showed a gradual but nonsignificant decrease in the number of migrants (ANOVA, *p* > .05) between DHC, HC, and I systems in *C. mucedo*. The proportions of migrants in populations from DHC sites varied between 0.25 (EST) and 1 (WRO, WDN, BIS); in DH sites from 0.14 (LOL and BAS) to 1 (BTF and DRO); and in I sites between 0 (SEL, BRY, CGN) and 1 (BAR and MOK). In the case of *F. sultana*, there was a nonsignificant trend for numbers of migrants to be larger in populations from isolated sites than in DHC sites (ANOVA, *p* > .05). The proportions of migrants in populations from DHC sites varied from 0.14 (WIM) to 1 (SBK, RBL); in I systems varied between 0.18 (GLA) and 1 (STI, WAV, YEW, BLW, LUW).

### 3.4 Spatial patterns of genetic diversity

Patterns of clonal diversity show regional variation for *F. sultana* with greater clonal diversity (*N*_C_: Kruskal–Wallis, *p* < .001) and richness (*R*: ANOVA, *p* < .001) in Cumbria relative to Norfolk and GG‐NI (Greater Glasgow–Northern Ireland; Figure 4; Table S4). In *C. mucedo* populations, *F*_IS_ was significantly lower in Norfolk and Cumbria than in GG‐NI (ANOVA, *p* < .05). There was no evidence of regional variation in other estimates of genetic diversity or migration in *C. mucedo* and *F. sultana* populations (ANOVA tests all with *p* > .05; Table S4).

**Figure 6:**
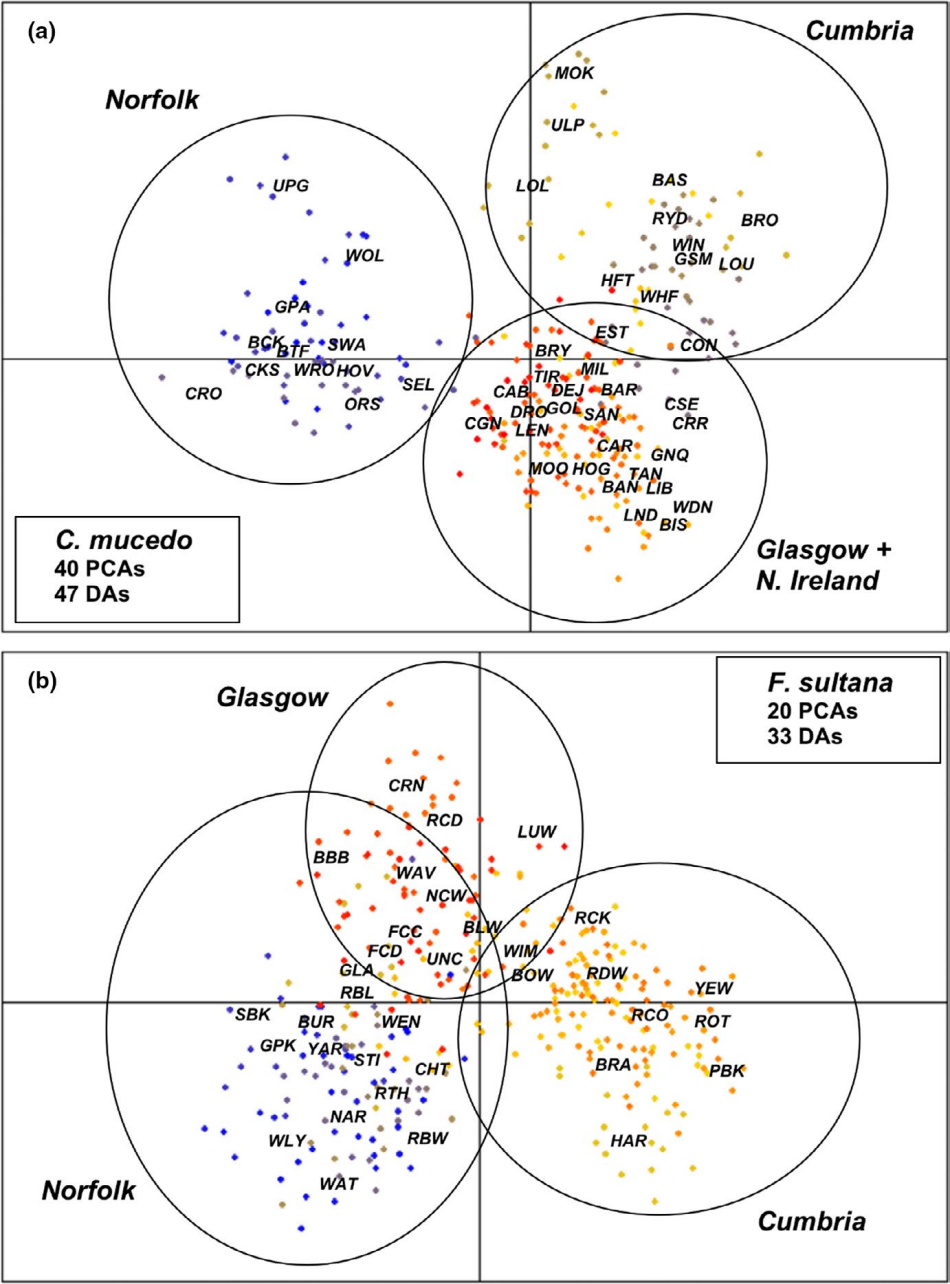
General population structure according to DAPC plots for *Cristatella mucedo* (a) and *Fredericella sultana* (b). Dot colors reflect the degree of genetic relatedness among samples. Circles outline the main genetic clusters in geographical region (i.e., Norfolk, Cumbria, Glasgow, and Northern Ireland). Boxes provide details for the number of PCAs and significant discriminant axes (DAs) retained included in each plot

The general discriminant analysis of principle components (DAPC) resolved *C. mucedo* MLGs as three major genetic clusters: (a) Norfolk populations, (b) Cumbria populations, and (c) Glasgow and Northern Ireland populations (variance explained along the main [horizontal] axis = 88.30%; Figure 6). The first DA largely separated the Norfolk cluster from all the other populations, while the second DA separated the cluster containing Glasgow + Northern Ireland populations from the remaining populations. DAPC analysis of *F. sultana* genotypes defined three major clusters: (a) Norfolk populations, (b) Cumbria populations, and (c) Glasgow populations (variance explained along the main axis = 84.73%; Figure 6). The first DA largely separated the Cumbria cluster from all other populations, while the second DA separated the Glasgow cluster from the remaining populations. The independent STRUCTURE analyses (Figure S1) highlighted a main genetic structure for two populations for *C. mucedo* (*K* = 2, Ln *P*(*K*) = −10,203.18 ± 1.94) that were characterized by the Norfolk populations and the remaining material (Figure S1). An additional likely substructure was detected as: (a) Norfolk, (b) Cumbria, and (c) Glasgow + Northern Ireland populations (*K* = 3, Ln *P*(*K*) = −9,585.90 ± 3.70; Figure S1). STRUCTURE analysis of the *F. sultana* material (Figure S1) identified a most likely occurrence of two clusters that were characterized by the Cumbrian populations and those from Norfolk and Glasgow (*K* = 2, Ln *P*(*K*) = −12,222.26 ± 3.19; Figure S1).

The assignment test with GENECLASS indicated that the number of migrants did not vary with regions in *C. mucedo* (ANOVA, *p* > .05). The proportion of migrants in Norfolk populations varied between 0 (SEL) and 1 (BTF and WRO), in Cumbria from 0.14 (LOL and BAS) to 1 (MOK), in Glasgow from 0.14 (CRR) to 1 (BAR, BIS and WDN), and in Northern Ireland from 0 (BRY and CGN) to 1 (DRO). There was a trend for the proportion of migrants in *F. sultana* populations to be larger in Norfolk and in Glasgow than in Cumbria (ANOVA, *p* > .05). The proportion of migrants in Norfolk populations varied between 0.18 (GLA) and 1 (SBK, RBL, STI and WAV), in Cumbria from 0.14 (WIM) to 1 (YEW), and in Glasgow from 0.31 (UNC) to 1 (BLW and LUW).

### 3.5 Evidence for genetic divergence, selection and bottlenecks

Mantel tests revealed a lack of correlation (*p* > .05) between geographic distance and *F*_ST_ values for both *C. mucedo* and *F. sultana* populations within Norfolk, Glasgow, and Northern Ireland (Figure S2). However, in both cases *F*_ST_ and geographic distance were significantly correlated in Cumbria (*C. mucedo*: *p* < .001, *F. sultana*: *p* < .01; Figure S2).

In our analyses for neutrality, 16 of 66 tests to detect outliers in the *F. sultana* datasets were significant (HIM test in Arlequin, *p* < .05; Table 1a). Following Bonferroni correction, three tests remained significant (locus Fs14TKUfor overall data; locus Fs14TKU for Norfolk vs. Cumbria data; locus Fs04 for populations in isolated vs. DHC sites in Cumbria). Six of 63 tests of the *C. mucedo* datasets (*F*_ST_‐based test in Arlequin) were significant (*p* < .05) but none remained significant after the Bonferroni correction for multiple tests (Table 1). The Jeffrey's metric for identifying loci under selection in Bayescan2.1 provided evidence of selection acting on 3 out of 10 and 1 out of 10 loci for *F. sultana* and *C. mucedo*, respectively (Table 1). The only locus that was not in HW equilibrium and was identified as a candidate outlier (thus potentially under selection) is Fsu04 in *F. sultana*. Excluding this locus from the general analysis performed did not change the results obtained. Hence, it was retained in the final dataset here used. In general, loci found in *F. sultana* as candidate outliers were showing sign of LD that would reinforce the idea that non‐neutral processes mediated by local selection are in action for *F. sultana* populations.

Thirty‐four of 48 populations (70.8%) of *C. mucedo* and 26 of 34 populations (76.5%) of *F. sultana* were characterized by a sufficient number of MLGs (≥4) to conduct BOTTLENECK 1.2 tests. Thirteen of 26 tests (50%) and 20 of 34 tests (58.8%) provided evidence for recent bottlenecks in *F. sultana* and *C. mucedo*, respectively (Table S5). A higher proportion of such bottlenecked populations were from connected sites for *C. mucedo* (70%; based on 4 DHC, 10 HC and 6 isolated sites) than for *F. sultana* (33%; based on 4 connected and 8 isolated sites).

## 4 DISCUSSION

### 4.1 Dispersal capacity, genetic diversity, and gene flow

Our results provide strong evidence that genetic diversity and gene flow in populations of *C. mucedo* are influenced by hydrological connectivity. Thus, shared multilocus genotypes (MLGs) were disproportionately detected among populations in directly hydrologically connected (DHC) sites (14 of 15 cases). Furthermore, populations in 10 of the 11 DHC sites were characterized by ongoing gene flow while there was no evidence for gene flow among populations in the 26 sites that were not directly hydrologically connected. There were also greater numbers of clones, alleles, and migrants in populations from DHC sites and higher values for clonal richness and expected heterozygosity in these populations. Discriminant analysis of principal components (DAPC) separated populations according to hydrological connectivity in Cumbria and Norfolk. The failure of DAPC to separate populations from Greater Glasgow and Northern Ireland according to our measures of hydrological connectivity suggests other complicating processes. For example, sites in Northern Ireland can be periodically connected during occasional seasonal flooding (Salgado et al., 2018) while movements of waterbirds, including ducks, swan, and geese, link the two areas encompassing these populations (Scott & Rose, 1996). Genetic similarity of populations in these two regions could be promoted if statoblasts are transported by bird movements.

Overall, our results provide collective evidence that drifting *C. mucedo* floatoblasts relatively frequently enable colonization within hydrologically connected systems. We thus demonstrate that hydrochory in addition to zoochory influences population genetic structure in this species. The maximum dispersal distance that our data suggest is effected by hydrochory is roughly 22 km (based on identical clonal genotypes collected in Grasmere and Windermere). However, we would expect that hydrochory could be achieved across much greater distances given the extensive distances associated with some hydrologically connected systems and diapause through the entire winter period of *C. mucedo* statoblasts. Dispersal by other means may also contribute to the observed patterns. For example, statoblasts may be disseminated by animal vectors that either require hydrological connectivity (e.g., fish) to reach distant sites or that may be prone to follow water courses (e.g., humans, other mammals or birds). Notably *C. mucedo* statoblasts are often observed in digestive tracts of fish and waterbirds and in waterbird feces, and they have been shown to hatch after passage through vertebrate digestive tracts (Okamura et al., 2019). Rafting of colonies could also contribute to gene flow. However, delicate gelatinous *C. mucedo* colonies are restricted to microhabitats offering protection from flow. The contribution of rafting colonies to gene flow is thus likely to be low relative to that conferred by statoblasts which are produced in large numbers and whose small size and external hardened valves protect them from damage.

Notably there were no shared MLGs between landscapes. This contrasts with evidence of identical *C. mucedo* genotypes in sites in northern Europe separated by some 700 km of land and sea (Freeland et al., 2000). These contrasting results may be explained by greater genetic resolution provided by a larger number of microsatellite loci in our study (10 instead of 5) and/or poor resolution based on few microsatellites. The latter, however, was not supported by a simulation analysis based on random assignments of clones (Freeland et al., 2000). Alternatively, they may be explained by the regular, long‐distance movements of migratory waterbirds that link the sites across northwest Europe studied by Freeland et al. (2000). Such migratory movements generally were not associated with sites in our study which was designed to assess the influence of hydrological connectivity within landscapes. However, as noted above, waterbird movements may have linked populations in Greater Glasgow and Northern Ireland. The detection of identical MLGs in two isolated sites separated by ~1 km of linear distance in Cumbria is also indicative of dispersal not mediated by hydrological connectivity. The common distribution of this clone could be explained by local movements of more sedentary waterbirds or human‐mediated transport.

In strong contrast, there was relatively little evidence that genetic diversity and gene flow in populations of *F. sultana* are influenced by hydrological connectivity. There were only three cases of shared clones among sites—twice between sites on the same river (River Carron in Greater Glasgow) and once between sites in different but relatively close river catchments in Norfolk (the Rivers Stiffkey and Wensum whose watersheds and associated ditch networks are in places separated by <2 km). Hydrological connectivity had no impact on any measures of population genetic diversity or gene flow. These results suggest that piptoblasts are effective at routinely remaining within the vicinity where they were produced, contributing to persistence within local populations and limiting downstream dispersal that could invariably lead to the sea. It also suggests that, despite a propensity for the tubular, branching colonies to fragment and re‐attach to surfaces (Fontes, Hartikainen, Taylor, et al., 2017; Wood, 1973), detached branches of *F. sultana* may not be spread regularly downstream to become established in distant sites. Instead, idiosyncratic deposition of drifting branches may result from interactions of geomorphology and flow and populations arising from such colonization were simply not sampled.

Patterns of genetic diversity in *F. sultana* populations varied more among regions than those of *C. mucedo*. In particular, clonal diversity, clonal richness, and number of alleles were highest in Cumbria suggesting that genetic diversity has accumulated over time in populations ranging over varying elevations in this more upland region. Alternatively, genetic diversity may have been lost in populations in Norfolk and Greater Glasgow, for example, due to recent selection regimes associated with substantial agricultural and urban development in Norfolk and Greater Glasgow, respectively. Evidence for lower inbreeding (*F*_IS_) of Cumbrian *C. mucedo* populations and positive correlations between *F*_ST_ and geographic distance for both species in Cumbria are somewhat consistent with either older upland populations in more varied sites or impacts of selection in the other regions. Finally, both DAPC and STRUCTURE analyses resolved all *F. sultana* populations according to their respective regions of collection.

### 4.2 Dispersal capacity and genetic divergence

Our results provide evidence that varying dispersal capacities of *C. mucedo* and *F. sultana* modulate genetic variation within and among benthic invertebrate populations, and thus, how life‐history traits may be linked with genetic divergence and the potential for local selection. Evidence for a relatively greater impact of these processes in *F. sultana* populations includes a greater number of loci potentially under selection (5 vs. 1 outlier out of 10 loci) and more cases of linkage disequilibrium between loci (2 vs. 0) for *F. sultana* and *C. mucedo*, respectively. The inference of greater genetic divergence in *F. sultana* is further supported by the larger number of unique clones detected overall (despite a smaller number of samples that were genotyped), as well as the greater average clonal diversity and greater average genotype richness values within sites for *F. sultana* relative to *C. mucedo* samples.

There was a somewhat higher proportion of *C. mucedo* populations with evidence of bottlenecking than in *F. sultana* (58.8% and 46.2%, respectively). This is in keeping with substantial evidence for metapopulation dynamics in *C. mucedo* in the form of extinction and colonization (Okamura, 1997; Okamura et al., 2013) and gene flow (Freeland et al., 2000). Relatively similar proportions of bottlenecking in *F. sultana* populations support metapopulation dynamics. However, greater genetic divergence suggests slower dynamics—an inference in keeping with the lower proportion of bottlenecked populations in hydrologically connected sites in *F. sultana* than in *C. mucedo* (33% and 70%, respectively).

The above arguments suggest that general lack of conformation to Hardy–Weinberg equilibrium in both species may be explained by founder effects and selection in *F. sultana* and bottlenecks in *C. mucedo* populations. Further independent evidence in support of the contrasting patterns of genetic variation in these two species is obtained from analyses of mtDNA variation in populations from Switzerland and the UK (Okamura et al., 2019). Analysis of molecular variance (AMOVA) demonstrated genetic divergence between populations from the two countries for *F. sultana* but not for *C. mucedo*. *F. sultana* also exhibited a greater number of haplotypes than *C. mucedo*, and a significantly greater proportion of haplotypes was unique to populations of *F. sultana*.

### 4.3 Comparisons with other passively dispersed aquatic invertebrates

Patterns of genetic variation revealed in our study compared with those of other passively dispersing aquatic organisms provide insights on how life‐history traits may influence genetic diversity. Many highly dispersive taxa are paradoxically linked with substantial levels of genetic differentiation even when populations are in close proximity—a pattern that led to the development of the widely recognized “Monopolization Hypothesis” (De Meester et al., 2002). The hypothesis proposes that rapid population growth and local adaptation following colonization of sites by cyclically parthenogenetic zooplankton (cladocerans, rotifers) preclude successful establishment of incoming, less relatively well‐adapted colonists. Hence, under this scenario the maintenance of genetic divergence among zooplankton populations is explained by a strong priority effect. The establishment of dormant egg banks of locally adapted zooplankton progeny is anticipated to further enable effective monopolization of resources. Support for such processes includes evidence for rapid local adaptation in zooplankton (e.g., Cousyn et al., 2001; Decaestecker et al., 2007), persistent founder effects in zooplankton populations (Boileau, Hebert, & Schwartz, 1992; Gómez, Adcock, Lunt, & Carvalho, 2002), and the development of egg banks in sediments (Brendonck & De Meester, 2003). Recent simulation modeling also demonstrates that persistent founder effects can strongly impact population structure of highly dispersive zooplankton given high population growth rates, large population size, and the presence of diapausing egg banks (Montero‐Pau, Gómez, & Serra, 2018). During early stages of colonization, however, there may be a window of time when ongoing migration could be favoured. For example, in young metapopulations of *Daphnia* outbred genotypes were found to increase in frequency due to hybrid vigor (Ebert et al., 2002). Clearly, the lead‐in time to monopolization will variously depend on rates of dispersal, population growth, local adaptation, and egg bank development. Accordingly, Badosa et al. (2017) showed that external source populations and residual egg banks led to persistent founder effects in some rotifer populations but not others. Patterns and rates of extinction due to abiotic drivers or disease may also be influential. These processes are likely to vary greatly among taxa and environments and to such an extent that monopolization may in some cases rarely, if ever, be achieved despite a general propensity for monopolization of resources to develop in zooplankton taxa.

*Cristatella mucedo* provides a contrasting model system for aquatic invertebrates. In common with zooplankton, *C. mucedo* has a high capacity for passive dispersal, but unlike zooplankton, populations are not prone to monopolization effects. Thus, there is consistent evidence for considerable ongoing gene flow among populations mediated by hydrological connectivity (this study) and migratory waterbirds (Figuerola et al., 2005; Freeland et al., 2000). A key trait is the production of buoyant dormant dispersive stages (statoblasts) that can survive passage through digestive tracts and may be carried to distant sites in hydrologically connected systems by prevailing currents. Bottlenecking (evidence from this study), infection by myxozoan (Okamura, 1996) and microsporidian (Canning, Refardt, Vossbrinck, Okamura, & Curry, 2002) parasites, and intense predation pressure (B. Okamura, unpublished data) are potential sources of local extinction. Furthermore, although *C. mucedo* shares extensive clonal reproduction with cyclically parthenogenetic zooplankton, unlike zooplankton, dormant statoblasts are not sexual products. Local adaptation in *C. mucedo* could thus be constrained because genetic variation introduced by sex is only generated during a brief period early in the growing season (Okamura & Freeland, 2002). This prediction gains some support from the lack of evidence for selection in this study. It is likely that statoblasts produced in the previous year are largely responsible for the development of populations in the following late spring/early summer, but genetic evidence suggests that older statoblasts present in statoblast banks in sediments can also occasionally contribute to population genetic diversity and decrease the likelihood of local extinction (Freeland, Rimmer, & Okamura, 2001).

Our results for *F. sultana* populations contribute a more nuanced and well‐rounded picture of the relative importance of processes influencing genetic variation among populations of passively dispersing invertebrates. In this case, genetic differentiation and divergence of *F. sultana* populations are linked with low gene flow as might be expected. However, these processes in *F. sultana* are not associated with large dormant egg banks in sediments as in zooplankton systems, but with the production of nondispersive, asexually produced propagules that remain attached to surfaces. Callaghan (1996) found that asynchronous germination of similarly attached *Plumatella emarginata* statoblasts (sessoblasts) enhances survivorship. This suggests that dormancy variation can influence postrecruitment dynamics at least in some bryozoan systems. However, the extent that these surface‐associated propagules can effectively act like a zooplankton “egg bank,” enhancing persistence of locally adapted genotypes and thwarting colonization, requires further study.

In summary, our two bryozoan systems and cyclically parthenogenetic zooplankton collectively illustrate a range of demographic and evolutionary processes reflecting particular life‐history attributes of aquatic invertebrates. These outcomes include the following: (a) genetic divergence among populations in association with low dispersal capacity (*F. sultana*), (b) ongoing gene flow and relatively low genetic differentiation among populations associated with high dispersal capacity (*C. mucedo*), and (c) genetic differentiation and local adaptation in populations despite high dispersal capacity due to rapid population growth, large propagule banks and persistent founder effects (cyclically parthenogenetic zooplankton).

### 4.4 Aquatic biodiversity and conservation

Our study provides evidence that propagule retention promotes genetic divergence among *F. sultana* populations. Such retention is likely to reduce displacement downstream and is characteristic of sponge gemmules, bryozoan piptoblasts and sessoblasts, and embryos of *Hydra*. Propagule retention may thus be a critical life‐history trait that enables many attached forms to inhabit running water systems rather than being swept to the sea. Exceptions that prove the rule include glochidia larvae that are released by bivalves to hitchhike on fish in order to colonize new sites. Due to the constraints imposed by flow and displacement, river habitats may particularly promote population divergence of inhabitants that lack mobility in general, or at critical periods in their life history. Indeed, this scenario of constraint may fundamentally explain the numerous subspecies, strains, and lineages of salmonids whose spawned eggs are retained in specific stretches of particular rivers in the Pacific coast of North America (e.g., Johnson, Kemp, & Thorgaard, 2018; Penaluna et al., 2016). Additional factors may also influence rates of evolution in lotic environments. For example, Fujisawa, Vogler, and Barraclough (2015) provide evidence that faster rates of evolution in lotic than lentic water beetles may be related to smaller population sizes in lotic environments. In addition, spatially explicit agent‐based modeling suggests that complex architectures and downstream flow regimes of rivers promote genetic diversity (Thomaz et al., 2016). While it is apparent that ancient isolation of large persistent lakes has been accompanied by unique radiations (e.g., sponges in Lake Baikal; cichlids in the African Rift Valley Lakes), the diverse physical environments offered by rivers may particularly support divergence and speciation. The unique conditions and demands of running water systems may therefore explain why riverine fishes disproportionately contribute to global vertebrate diversity (Thomaz et al., 2016).

Spatial and temporal patterns of dispersal and colonization will contribute to biodiversity and species distributions and abundances. In turn, variation in life‐history traits, such as dispersal capacity and population growth rates, can determine the extent that populations diverge and may therefore resist invasion by externally sourced colonists. Taxa associated with running water habitats and with limited gene flow among populations, such as *F. sultana*, may thus be most impacted by the many changing conditions that now commonly challenge our biota, such as habitat loss, agricultural intensification, climate change, and invasive species. In contrast, taxa with high dispersal capacity and regular gene flow, such as *C. mucedo* and zooplankton, may locate favorable environments and, in the case of zooplankton, may rapidly adapt to new conditions. These considerations imply that conservation of river inhabitants is of particular concern in our changing world.

## CONFLICT OF INTEREST

None declared.

## AUTHOR CONTRIBUTIONS

This research was designed by BO, who also contributed in the collection of samples, interpretation of results and development of the manuscript. PR contributed in the collection of samples, DNA extractions, genotyping, computational analyses, and the development of the manuscript. EP helped in collecting *Cristatella mucedo* statoblasts from Northern Ireland sediments.

## Data Availability

The datasets of genotyped individuals of *Cristatella mucedo* and *Fredericella sultana* are available in Dryad (https://doi.org/10.5061/dryad.1tm8705) enabling anyone to replicate our analyses.
