# Supplementary material for: To remain or leave: Dispersal variation and its genetic consequences in benthic freshwater invertebrates
Source: Ecol Evol. 2019 Oct 18;9(21):12069–88. doi: 10.1002/ece3.5656 (PMC6854113; doi:10.1002/ece3.5656)
Supplement: Supplementary file 1 [file ECE3-9-12069-s001.pdf]

**Table S1.** Summary of material subject to genetic analysis providing information on name and type of locality ([L] = lake; [R] = river; [C] = canal) (and catchment for localities on rivers); site code as located in Fig. 1 (Site code); hydrological connectivity (HC) type as DHC (directly hydrologically connected), HC (hydrologically connected), C (connected), I (isolated) (see methods for further details); latitude (Lat) and longitude (Long); date of collection, sample size (N), and population genetic summary statistics. $N_C$ = number of unique clones per sample; $R$ = genotypic richness; $H_E$ = expected heterozygosity; $H_O$ = observed heterozygosity; $N_A$ = mean number of alleles per sample; $F_{IS}$ = inbreeding coefficient index.

| **Locality (catchment)** | **Site code** | **HC-type** | **Lat (°N)** | **Long (°E)** | **N** | **Date** | $N_C$ | $R$ | $H_E$ | $H_O$ | $N_A$ | $F_{IS}$ |
|---|---|---|---|---|---|---|---|---|---|---|---|---|
| **NORFOLK** | | | | | | | | | | | | |
| ***C.mucedo*** | | | | | | | | | | | | |
| Cockshoot Broad [L] | CKS/1 | DHC | 52.6886 | 1.4678 | 34 | 10/05/16 | 8 | 0.21 | 0.640±0.041 | 0.725±0.050 | 3.40±1.07 | -0.144 |
| Hoveton Great Broad [L] | HOV/2 | DHC | 52.6928 | 1.4332 | 36 | 07/10/15 | 10 | 0.26 | 0.728±0.017 | 0.687±0.050 | 4.80±1.14 | 0.062 |
| Inner South Walsham Broad [L] | SWA/3 | DHC | 52.6720 | 1.4956 | 25 | 14/03/15 | 8 | 0.29 | 0.710±0.049 | 0.754±0.050 | 5.00±1.33 | -0.066 |
| Wroxham Broad [L] | WRO/4 | DHC | 52.6987 | 1.4173 | 28 | 0710/16 | 4 | 0.11 | 0.793±0.022 | 0.750±0.069 | 3.90±0.74 | 0.063 |
| Burntfen Broad [L] | BTF/5 | HC | 52.7130 | 1.4610 | 30 | 12/05/16 | 2 | 0.03 | 0.667±0.043 | 0.800±0.089 | 2.30±0.48 | -0.333 |
| Crome's South Broad [L] | CRO/6 | HC | 52.7222 | 1.5130 | 25 | 05/10/16 | 5 | 0.17 | 0.603±0.057 | 0.630±0.069 | 3.10±1.29 | -0.049 |
| Ormesby Broad (North) [L] | ORS/7 | HC | 52.6791 | 1.6430 | 38 | 11/05/16 | 11 | 0.27 | 0.706±0.039 | 0.707±0.047 | 4.10±1.37 | 0.022 |
| Upton Great Broad [L] | UPG/8 | HC | 52.6650 | 1.5309 | 23 | 04/10/16 | 8 | 0.32 | 0.489±0.068 | 0.430±0.056 | 2.80±1.23 | 0.128 |
| Buckenham Broad [L] | BCK/9 | I | 52.5969 | 1.4865 | 31 | 15/03/15 | 3 | 0.07 | 0.693±0.052 | 0.600±0.089 | 3.10±0.74 | 0.163 |
| Sawmill Pond - Gunton Park [L] | GPA/10 | I | 52.8531 | 1.3011 | 30 | 11/05/16 | 4 | 0.09 | 0.653±0.053 | 0.683±0.076 | 3.00±0.94 | -0.058 |
| Selbrigg Pond [L] | SEL/11 | I | 52.9069 | 1.1323 | 33 | 0810/16 | 2 | 0.03 | 0.833±0.035 | 0.700±0.103 | 3.00±0.67 | 0.222 |
| Wolterton [L] | WOL/12 | I | 52.8359 | 1.2105 | 32 | 03/10/16 | 4 | 0.10 | 0.617±0.036 | 0.675±0.075 | 2.60±0.52 | -0.117 |
| ***F.sultana*** | | | | | | | | | | | | |
| Blackwater (Wensum) [R] | RBW/13 | C | 52.7375 | 1.0910 | 34 | 01/07/16 | 9 | 0.24 | 0.690±0.039 | 0.711±0.051 | 4.40±1.51 | -0.028 |
| R Blackwater (Yare) [R] | RBL/14 | C | 52.6103 | 0.9938 | 26 | 02/07/16 | 1 | 0.00 | 0.700±0.153 | 0.700±0.142 | 1.70±0.48 | NA |
| R Bure (Bure) [R] | BUR/15 | C | 52.8294 | 1.2144 | 27 | 03/07/16 | 5 | 0.15 | 0.627±0.059 | 0.660±0.067 | 3.60±1.07 | -0.006 |
| R Chet (Yare) [R] | CHT/16 | C | 52.5414 | 1.4392 | 29 | 05/07/16 | 2 | 0.04 | 0.817±0.063 | 0.700±0.102 | 3.10±0.88 | 0.200 |
| R Wensum (Wensum) [R] | WEN/18 | C | 52.8371 | 0.8084 | 28 | 08/07/16 | 4 | 0.11 | 0.679±0.056 | 0.525±0.079 | 3.60±1.07 | 0.160 |
| Attlebridge (Wensum) [R] | WAT/19 | C | 52.7061 | 1.1484 | 30 | 13/07/16 | 15 | 0.48 | 0.671±0.048 | 0.655±0.043 | 5.10±1.91 | 0.015 |

| | | | | | | | | | | | | |
|---|---|---|---|---|---|---|---|---|---|---|---|---|
| Lyng (Wensum) [R] | WLY/20 | C | 52.7185 | 1.0658 | 24 | 13/07/16 | 15 | 0.61 | 0.706±0.049 | 0.640±0.040 | 6.10±2.02 | 0.098 |
| R Yare (Yare) [R] | YAR/21 | C | 52.6281 | 1.2342 | 32 | 16/07/16 | 12 | 0.35 | 0.683±0.044 | 0.672±0.045 | 5.10±1.52 | 0.021 |
| Scarrow Beck (Bure) [R] | SBK/22 | C | 52.8306 | 1.2347 | 24 | 16/07/16 | 1 | 0.00 | 0.556±0.176 | 0.556±0.166 | 1.56±0.53 | NA |
| R Stiffkey (Stiffkey) [R] | STI/17 | I | 52.8692 | 0.8469 | 35 | 08/07/16 | 1 | 0.00 | 0.600±0.163 | 0.600±0.155 | 1.60±0.52 | NA |
| R Glaven (Glaven) [R] | GLA/23 | I | 52.9046 | 1.1256 | 23 | 06/07/16 | 11 | 0.45 | 0.683±0.041 | 0.746±0.042 | 4.40±1.26 | -0.096 |
| R Nar (Nar) [R] | NAR/24 | I | 52.6984 | 0.6097 | 27 | 07/07/16 | 3 | 0.08 | 0.700±0.058 | 0.600±0.089 | 3.30±0.95 | 0.172 |
| R Thet (Thet) [R] | RTH/25 | I | 52.4417 | 0.9246 | 26 | 09/07/16 | 13 | 0.48 | 0.708±0.054 | 0.599±0.047 | 5.10±2.08 | 0.164 |
| R Waveney (Waveney) [R] | WAV/26 | I | 52.3671 | 1.1281 | 16 | 09/07/16 | 1 | 0.00 | 0.800±0.133 | 0.800±0.127 | 1.80±0.42 | NA |
| Sawmill Pond - Gunton Park [C] | GPK/27 | I | 52.8529 | 1.3012 | 30 | 01/07/16 | 9 | 0.28 | 0.635±0.044 | 0.667±0.050 | 3.60±1.07 | -0.054 |

| **Locality (catchment)** | **Site code** | **HC-type** | **Lat (°N)** | **Long (°E)** | **N** | **Date** | $N_C$ | $R$ | $H_E$ | $H_O$ | $N_A$ | $F_{IS}$ |
|---|---|---|---|---|---|---|---|---|---|---|---|---|
| **CUMBRIA** | | | | | | | | | | | | |
| ***C.mucedo*** | | | | | | | | | | | | |
| Esthwaite [L] | EST/28 | DHC | 54.3600 | -2.9902 | 30 | 06/07/17 | 8 | 0.24 | 0.815±0.029 | 0.529±0.064 | 5.20±1.75 | **0.394*** |
| Grasmere [L] | GSM/29 | DHC | 54.4539 | -3.0283 | 25 | 12/07/17 | 6 | 0.21 | 0.692±0.080 | 0.667±0.065 | 4.00±1.49 | 0.048 |
| Rydal Water [L] | RYD/30 | DHC | 54.4472 | -2.9880 | 25 | 28/06/16 | 8 | 0.29 | 0.715±0.034 | 0.657±0.057 | 4.33±1.32 | 0.086 |
| Windermere [L] | WIN/31 | DHC | 54.2880 | -2.9562 | 34 | 30/06/16 | 6 | 0.15 | 0.723±0.034 | 0.713±0.063 | 3.60±1.26 | 0.029 |
| Bassenthwaite [L] | BAS/32 | HC | 54.6767 | -3.2422 | 29 | 13/07/17 | 7 | 0.21 | 0.682±0.036 | 0.605±0.061 | 3.70±1.06 | 0.156 |
| Brothers Water [L] | BRO/33 | HC | 54.5088 | -2.9251 | 27 | 30/05/17 | 6 | 0.19 | 0.648±0.054 | 0.693±0.060 | 3.70±1.25 | -0.076 |
| Coniston [L] | CON/34 | HC | 54.3661 | -3.0621 | 31 | 01/07/17 | 15 | 0.47 | 0.630±0.064 | 0.656±0.044 | 4.44±1.42 | -0.041 |
| Longlands Lake [L] | LOL/35 | HC | 54.5008 | -3.5287 | 28 | 21/05/17 | 7 | 0.22 | 0.708±0.033 | 0.662±0.060 | 3.90±0.74 | 0.073 |
| Loughrigg Tarn [L] | LOU/36 | HC | 54.4304 | -3.0090 | 30 | 27/05/17 | 4 | 0.10 | 0.688±0.072 | 0.750±0.077 | 2.80±1.03 | -0.130 |
| High Thorn Fish Farm [L] | HTF/37 | I | 54.3812 | -2.7221 | 30 | 15/07/17 | 2 | 0.03 | 0.433±0.100 | 0.500±0.118 | 1.80±0.63 | -0.250 |
| Mockerkin Tarn [L] | MOK/38 | I | 54.5947 | -3.4208 | 29 | 20/07/17 | 5 | 0.14 | 0.569±0.041 | 0.700±0.065 | 2.70±0.48 | -0.267 |
| Ullock Pond [L] | ULP/39 | I | 54.6044 | -3.4366 | 33 | 20/07/17 | 3 | 0.06 | 0.527±0.101 | 0.567±0.095 | 2.40±1.07 | -0.097 |
| Whinfell Tarn [L] | WHF/40 | I | 54.3756 | -2.6815 | 28 | 15/07/17 | 2 | 0.04 | 0.467±0.133 | 0.550±0.121 | 1.90±0.88 | -0.385 |
| ***F.sultana*** | | | | | | | | | | | | |
| Skelwith Bridge (R Brathay/Rothay) [R] | BRA/41 | C | 54.4209 | -3.0125 | 43 | 18/07/17 | 23 | 0.52 | 0.664±0.056 | 0.641±0.033 | 6.50±3.06 | 0.037 |
| Low Lorton (R Derwent/Cocker) [R] | RCO/42 | C | 54.6148 | -3.3177 | 27 | 26/07/17 | 16 | 0.58 | 0.632±0.065 | 0.556±0.042 | 5.80±3.43 | 0.129 |

| | | | | | | | | | | | | |
|---|---|---|---|---|---|---|---|---|---|---|---|---|
| Isel Bridge (R Derwent/Cocker) [R] | RDW/43 | C | 54.6878 | -3.2999 | 30 | 24/07/17 | 20 | 0.66 | 0.633±0.081 | 0.572±0.037 | 6.20±3.39 | 0.101 |
| Pelter Bridge (R Brathay/Rothay) [R] | ROT/44 | C | 54.4454 | -2.9807 | 30 | 18/07/17 | 13 | 0.41 | 0.645±0.040 | 0.532±0.046 | 4.80±1.69 | 0.191 |
| Bowness marina (R Brathay/Rothay) [L] | BOW/45 | C | 54.3604 | -2.9310 | 19 | 14/10/17 | 12 | 0.61 | 0.749±0.041 | 0.593±0.048 | 6.10±1.20 | **0.222*** |
| North Bowness (R Brathay/Rothay) [L] | WIM/46 | C | 54.4088 | -2.9506 | 31 | 14/10/17 | 21 | 0.67 | 0.759±0.027 | 0.650±0.035 | 6.70±1.25 | **0.148*** |
| Haweswater Reservoir outlet [R] | HAR/47 | I | 54.5361 | -2.7449 | 29 | 21/07/17 | 16 | 0.54 | 0.473±0.105 | 0.430±0.040 | 4.30±2.67 | 0.096 |
| Park Beck (input from Loweswater) [R] | PBK/48 | I | 54.5715 | -3.3271 | 36 | 24/07/17 | 13 | 0.34 | 0.523±0.089 | 0.461±0.047 | 4.10±2.13 | 0.135 |
| Spark Bridge (R Crake) [R] | RCK/49 | I | 54.2524 | -3.0667 | 23 | 17/07/17 | 16 | 0.68 | 0.657±0.072 | 0.511±0.042 | 5.30±2.00 | 0.235 |
| Yew Tree Tarn outflow [R] | YEW/50 | I | 54.3927 | -3.0472 | 28 | 15/10/17 | 12 | 0.41 | 0.464±0.063 | 0.501±0.047 | 2.70±0.48 | -0.082 |

| **Locality (catchment)** | **Site code** | **HC-type** | **Lat (°N)** | **Long (°E)** | **N** | **Date** | $N_C$ | $R$ | $H_E$ | $H_O$ | $N_A$ | $F_{IS}$ |
|---|---|---|---|---|---|---|---|---|---|---|---|---|
| **GREATER GLASGOW** | | | | | | | | | | | | |
| ***C. mucedo*** | | | | | | | | | | | | |
| Bishop Loch [L] | BIS/51 | DHC | 55.8762 | -4.0981 | 18 | 23/07/16 | 2 | 0.06 | 0.783±0.056 | 0.700±0.105 | 2.70±0.67 | 0.172 |
| Lochend [L] | LND/52 | DHC | 55.8723 | -4.0676 | 31 | 10/07/17 | 4 | 0.10 | 0.664±0.071 | 0.525±0.079 | 3.60±1.26 | 0.236 |
| Woodend Loch [L] | WDN/53 | DHC | 55.8765 | -4.0694 | 30 | 24/07/16 | 3 | 0.07 | 0.713±0.060 | 0.467±0.094 | 2.90±0.74 | 0.457 |
| Carbeth Loch [L] | CAR/54 | HC | 55.9847 | -4.3503 | 30 | 10/07/17 | 9 | 0.28 | 0.659±0.037 | 0.572±0.057 | 3.20±0.79 | 0.150 |
| Castle Semple [L] | CSE/55 | HC | 55.7930 | -4.6212 | 34 | 25/07/16 | 5 | 0.12 | 0.723±0.041 | 0.620±0.072 | 4.10±1.10 | 0.159 |
| Garnqueen Loch [L] | GNQ/56 | HC | 55.8940 | -4.0521 | 29 | 10/07/17 | 4 | 0.11 | 0.714±0.084 | 0.633±0.077 | 3.80±1.32 | 0.131 |
| Hogganfield Park Loch [L] | HOG/57 | HC | 55.8799 | -4.1725 | 24 | 22/07/16 | 5 | 0.17 | 0.674±0.063 | 0.577±0.077 | 3.80±1.23 | 0.181 |
| Tannoch Loch [L] | TAN/58 | HC | 55.9491 | -4.3142 | 29 | 08/07/17 | 4 | 0.11 | 0.540±0.078 | 0.617±0.081 | 2.40±0.84 | -0.175 |
| Banton Loch [L] | BAN/59 | I | 55.9829 | -4.0304 | 23 | 17/05/17 | 7 | 0.27 | 0.654±0.081 | 0.575±0.063 | 4.44±1.59 | 0.131 |
| Bardowie Loch [L] | BAR/60 | I | 55.9359 | -4.2809 | 28 | 27/07/16 | 5 | 0.15 | 0.761±0.054 | 0.503±0.080 | 4.30±1.49 | **0.372*** |
| Carron Valley Reservoir [L] | CRR/61 | I | 56.0309 | -4.0617 | 27 | 12/07/17 | 7 | 0.23 | 0.704±0.034 | 0.760±0.053 | 4.20±1.23 | -0.088 |
| Libo Loch [L] | LIB/62 | I | 55.7699 | -4.4951 | 28 | 26/07/16 | 9 | 0.30 | 0.667±0.048 | 0.633±0.054 | 4.20±1.03 | 0.057 |
| ***F.sultana*** | | | | | | | | | | | | |
| Forth Clyde Canal (Site 1) [C] | FCC/63 | C | 55.9720 | -4.0253 | 40 | 11/07/17 | 11 | 0.26 | 0.791±0.031 | 0.604±0.049 | 6.50±1.43 | **0.247*** |
| Forth Clyde Canal (Site 2) [C] | FCD/64 | C | 56.0025 | -3.8585 | 33 | 01/10/17 | 13 | 0.38 | 0.745±0.034 | 0.528±0.046 | 5.80±1.69 | **0.304*** |
| R Carron (Site 1) [R] | CRN/65 | C | 56.0316 | -4.0108 | 32 | 11/07/17 | 8 | 0.23 | 0.701±0.032 | 0.718±0.051 | 4.40±1.43 | -0.066 |
| R Carron (Site 2) [R] | RCD/66 | C | 56.0258 | -3.9206 | 25 | 12/07/17 | 10 | 0.38 | 0.710±0.049 | 0.618±0.050 | 5.00±1.76 | 0.140 |

| | | | | | | | | | | | | |
|---|---|---|---|---|---|---|---|---|---|---|---|---|
| Barbauchlaw Burn (Forrestburn) [R] | BBB/67 | I | 55.8649 | -3.8040 | 32 | 03/10/17 | 6 | 0.16 | 0.609±0.031 | 0.733±0.057 | 3.50±0.71 | -0.231 |
| Blane Water [R] | BLW/68 | I | 56.0098 | -4.3770 | 22 | 08/07/17 | 1 | 0.00 | 0.800±0.133 | 0.800±0.127 | 1.80±0.42 | NA |
| Luggie Water [R] | LUW/69 | I | 55.9291 | -4.0103 | 21 | 10/07/17 | 1 | 0.00 | 0.400±0.163 | 0.400±0.155 | 1.40±0.52 | NA |
| North Calder Water (Hillend Reserv.) [R] | NCW/70 | I | 55.8890 | -3.8755 | 35 | 02/10/17 | 13 | 0.35 | 0.700±0.036 | 0.579±0.046 | 4.10±1.66 | 0.192 |
| Union Canal (near Almond) [C] | UNC/71 | I | 55.9649 | -3.6628 | 19 | 04/10/17 | 13 | 0.67 | 0.829±0.029 | 0.575±0.048 | 8.20±2.39 | 0.318 |

| **Locality (catchment)** | **Site code** | **HC-type** | **Lat (°N)** | **Long (°E)** | **N** | **Date** | $N_C$ | $R$ | $H_E$ | $H_O$ | $N_A$ | $F_{IS}$ |
|---|---|---|---|---|---|---|---|---|---|---|---|---|
| **NORTHERN IRELAND** | | | | | | | | | | | | |
| ***C. mucedo*** | | | | | | | | | | | | |
| Carry Bridge, Upper Lough Erne [R] | CAB/72 | DHC | 54.284676 | -7.5477493 | 23 | 29/07/14 | 10 | 0.41 | 0.722±0.071 | 0.501±0.055 | 5.70±1.77 | **0.324*** |
| Derryad Jetty, Upper Lough Erne [R] | DEJ/73 | DHC | 54.193655 | -7.4797928 | 22 | 28/07/14 | 8 | 0.33 | 0.7691±0.093 | 0.498±0.068 | 3.90±1.79 | 0.362 |
| Tiraroe Jetty [R] | TIR/74 | DHC | 54.217234 | -7.5331554 | 26 | 01/08/14 | 11 | 0.40 | 0.690±0.059 | 0.624±0.049 | 4.90±1.52 | 0.100 |
| Gole Lough [L] | GOL/75 | HC | 54.292188 | -7.5475645 | 27 | 08/08/14 | 6 | 0.19 | 0.766±0.051 | 0.567±0.074 | 4.56±1.88 | 0.292 |
| Lough Drombominy [L] | DRO/76 | HC | 54.164983 | -7.4985267 | 22 | 28/07/14 | 3 | 0.10 | 0.593±0.131 | 0.556±0.104 | 2.33±1.12 | 0.122 |
| Mill Lough [L] | MIL/77 | HC | 54.140712 | -7.4957594 | 26 | 01/08/14 | 6 | 0.20 | 0.720±0.031 | 0.590±0.073 | 3.40±1.17 | 0.185 |
| Sand Lough [L] | SAN/78 | HC | 54.185318 | -7.4247477 | 29 | 30/07/14 | 9 | 0.29 | 0.706±0.060 | 0.536±0.058 | 4.70±2.31 | 0.262 |
| Barry Lough [L] | BRY/79 | I | 54.269888 | -7.5831296 | 26 | 07/08/14 | 4 | 0.12 | 0.649±0.077 | 0.725±0.077 | 2.80±1.03 | -0.192 |
| Cargin Lough [L] | CGN/80 | I | 54.197976 | -7.4408911 | 23 | 06/08/14 | 6 | 0.23 | 0.628±0.063 | 0.500±0.069 | 3.67±1.41 | 0.226 |
| Lehinch Lough [L] | LEN/81 | I | 54.187894 | -7.4001994 | 24 | 31/07/14 | 5 | 0.17 | 0.365±0.122 | 0.289±0.077 | 2.44±1.59 | 0.230 |
| Moorlough [L] | MOO/82 | I | 54.830784 | -7.3033333 | 26 | 04/08/14 | 2 | 0.04 | 0.600±0.094 | 0.500±0.112 | 2.40±0.97 | 0.231 |
