# Supplementary material for: To remain or leave: Dispersal variation and its genetic consequences in benthic freshwater invertebrates
Source: Ecol Evol. 2019 Oct 18;9(21):12069–88. doi: 10.1002/ece3.5656 (PMC6854113; doi:10.1002/ece3.5656)
Supplement: Supplementary file 2 [file ECE3-9-12069-s002.pdf]

Table S2. Site with shared clones for the two bryozoan species. Letters indicate distinct clones.

**Species**	**Sites**	**Clones**
Cristatella mucedo	Wroxham Broad-Cockshoot Broad	A
	Hoveton Broad-Cockshoot Broad	A,B,C
	Cockshoot Broad-South Walsham Broad	B
	Wroxham Broad-South Walsham Broad	B
	Wroxham Broad-Hoveton Broad	A
	Grasmere-Rydal Water	D E,F
	Grasmere-Windermere	G
	Bishop Loch-Woodend Lock	H
	Bishop Loch-Lochend	H
	Woodend Loch-Lochend	H
	Ullock Pond-Mockerkin Tarn	I
Fredericella sultana	River Wensum-River Stiffkey	J
	River Carron Site 1-River Carron Site 2	K,L
