# Supplementary material for: To remain or leave: Dispersal variation and its genetic consequences in benthic freshwater invertebrates
Source: Ecol Evol. 2019 Oct 18;9(21):12069–88. doi: 10.1002/ece3.5656 (PMC6854113; doi:10.1002/ece3.5656)
Supplement: Supplementary file 3 [file ECE3-9-12069-s003.pdf]

Table S3. Summary of the microsatellite loci, primers and PCR conditions used in the present study, including data on locus name, primer sequences, the number of repeat motifs, the annealing temperature (T_A), the fluorochrome used for primers label (Fluor.), the loci involved in the same multiplex (Multiplex), and reference to the development of the microsatellites and GenBank reference number (Microsat. development).

Locus Name	Primer Sequences	Repeat motifs	T_A	Fluor.	Multiple x	Species	*Microsat. development*
Cmu_2.5	F: AGTGCAAAGCCAGCTACCTAT	$(GA)_{27}$	60/55	6-FAM	A1	*C. mucedo*	*Unpublished*
	R: TTAGCAGCCAGTAGAGGCTATCG						*EU661819.1*
Cmu_9.4	F: ACAACAAACCCTTCAGCCAATG	$(GT)_{15}$	60/55	NED	A1	*C. mucedo*	*Freeland et al. 1999*
	R: ACAGTGGAGTTCAGCCAGTAATG						*AF085427.1*
Cmu_6.7	F: TACGACCCTACTGAATAACTCATGG	$(AG)_{13}$	60/55	NED	A1	*C. mucedo*	*Freeland et al. 1999*
	R: TTCCCTCGTGTCATTCACAG						*AF085424.1*
Cmu_2.3	F: AGGATCATATGCATTAGCACATATTGGGG	$(AC)_{11}$	60/55	6-FAM	A2	*C. mucedo*	*Unpublished*
	R: ACCGACCTCGTGAGACTTGAGAACTG						*EU661818.1*
Cmu_7.5	F: TCGAGAAACTGCTCTGCATCC	$(GA)_{18}$	60/55	VIC	A2	*C. mucedo*	*Unpublished*
	R: TCATCCGTCTGCACTTACCTTTATC						*EU661822.1*
Cmu_5.5	F: TCGTATGCATTTCTCGATGC		60/55	6-FAM	B1	*C. mucedo*	*Unpublished*
	F: CTCGTTCTGGACACCGGTAG						
Cmu_9.3	F: GGATCATAATAATAATAATGCACTTG	$(TC)_{24}$	60/55	PET	B1	*C. mucedo*	*Unpublished*
	R: ATACTATTATCTGCTGCGTCAGTC						*EU661823.1*
Cmu_3.6	F: ACCTGGTGATCTAACACGTCAC	$(TC)_{18}$	60/55	6-FAM	B1	*C. mucedo*	*Unpublished*
	R: TCAAAGAGACAGTGACAGCAGAAG						*EU661821.1*
Cmu_1.1	F: ATCGTGACATGGCTGACTTTAC	$(AG)_{32}$	60/55	VIC	B2	*C. mucedo*	*Freeland et al. 1999*
	R: AGCTACGTATGACGTTCTGCTC						*AF085418.1*
Cmu_2.2	F: GCAATTCTCACTTTCACTGTGCTC	$(CTGT)_7$ $(CTGT)_3$ $(CTGT)_5$ $(CT)_{51}$	60/55	PET	B2	*C. mucedo*	*Freeland et al. 1999*
	R: CTCGCGTCTGCCTGTCATTACA						*AF085420.1*
Fs09	F: CAACACATATGCGCACACAA	$(CA)_{18}$	62/57	6-FAM	A1	*F.sultana*	*Hartikainen and Jokela, 2011*
	R: *GCGAGACAGATGTGCAGGTA						
Fs09TKU	F: TCTAGCCGAGCCCAGACTAG	$(AGC)_{18}$	62/57	6-FAM	A1	*F.sultana*	*Filippenko et al., 2014*
	R: *CCTTTATGTGTGCCGAACCC						

Fs14TKU	F: CCAGGTTGGCACGCTCTATC<br>R: *GCAATACGGCGCTCTGAAG	$(ATC)_{13}$	62/57	Yakima Yellow	A1	*F.sultana*	*Filippenko et al., 2014*
Fs27	F: TTCAAACCATTACTAGCAGCCA<br>R: *CTCCATGTGACATCCGCTTA	$(GTAT)_{13}$	62/57	Yakima Yellow	A2	*F.sultana*	*Hartikainen and Jokela, 2011*
Fs24TKU	F: TCTCTCTAGCGTATCACCCAC<br>R: *GTTACGCGAGACAATGCAG	$(ATC)_{12}$	62/57	ATTO 565	A2	*F.sultana*	*Filippenko et al., 2014*
Fs13	F: GAGTGATGAGAGAGTGTCGGC<br>R: *CATCCGAACAACGCAGACTA	$(GT)_{16}$	62/57	6-FAM	B1	*F.sultana*	*Hartikainen and Jokela, 2011*
Fs12TKU	F: GCTCATGCGTCTTGACACTC<br>R: *AATGCAATCCGGATTTCGCTG	$(AGC)_{10}$	62/57	ATTO 565	B1	*F.sultana*	*Filippenko et al., 2014*
Fs25TKU	F: AGCAAGACATATAGAGCTGCAC<br>R: *ATGAATACCTGGCCGTCCTC	$(AGAT)_{15}$	62/57	6-FAM	B1	*F.sultana*	*Filippenko et al., 2014*
Fs04	F: CTCGCAGCTAATGTGGAACA<br>R: * CGCAAGAGAATGTCAGACCA	$(GT)_{18}$	62/57	Yakima Yellow	B2	*F.sultana*	*Hartikainen and Jokela, 2011*
Fs18	F: TCTCGAAGGCTACGTTCCAC<br>R: * GTTTGACCTTACCGCGCTC	$(TC)_{15}$	62/57	ATTO 550	B2	*F.sultana*	*Hartikainen and Jokela, 2011*

Filippenko, D., Hartikainen, H., Okamura, B., & Vasemagi, A. (2014). Characterisation of polymorphic microsatellite loci for the bryozoan *Fredericella sultana*, the primary host of the causative agent of salmonid proliferative kidney disease. *Conservation Genetics Resources*, 6(2), 481–482.

Freeland, J. R., Jones, C. S., Noble, L. R., & Okamura, B. (1999). Polymorphic microsatellite loci identified in the highly clonal freshwater bryozoan *Cristatella mucedo*. *Molecular Ecology Notes*, 8, 335–346.

Hartikainen, H. ,& Jokela, J. (2012). Characterisation of polymorphic microsatellite loci in the freshwater bryozoan *Fredericella sultana*. *Conservation Genetics Resources*, 4(2), 475–477.
