# Supplementary material for: To remain or leave: Dispersal variation and its genetic consequences in benthic freshwater invertebrates
Source: Ecol Evol. 2019 Oct 18;9(21):12069–88. doi: 10.1002/ece3.5656 (PMC6854113; doi:10.1002/ece3.5656)
Supplement: Supplementary file 4 [file ECE3-9-12069-s004.pdf]

Table S4. Patterns of genetic diversity and migration in *C. mucedo* and *F. sultana* populations collected from sites varying according to regions (Norfolk, Cumbria, GG = Greater Glasgow, NI = Northern Ireland) and hydrological connectivity (DHC = Directly Hydrologically Connected; HC = Hydrologically Connected; I = Isolated). (a) includes results of analyses when data for GG and NI were pooled. (b) includes results of analyses excluding data from NI. Values are means ± standard error. Bold values indicate cases where statistical tests indicated significant variation amongst populations according to connectivity or geographic regions. Superscripts associated with P-values indicate whether tests were ANOVA (A) or Kruskal-Wallis (K-W). NC = Number of clones; R = Genotypic richness index; H_E = expected heterogeneity; H_O = observed heterozygosity; NA = mean number of alleles; F_{IS} = inbreeding coefficient index; F_{ST} = genetic differentiation index (Weir & Cockerham 1984); M = historical migration rate (estimated by Migrate-n [M = m/m]; Beerli 2006, Beerli et al. 2009); m = present-day migrant percentage estimated by assignment test (GENECLASS 2.0; Rannala & Mountain 1997; Piry et al. 2004). ns = non-significant (P> 0.05); * = P< 0.05; ** = P< 0.01; *** = P<0.001.

(a)

C. mucedo

**Parameters**	***Norfolk***	***Cumbria***	***GG-NI***	***P-value***
N_C	5.75±0.90	6.08±0.94	5.83±0.53	ns ^{A}
R	0.16±0.03	0.18±0.03	0.19±0.02	ns ^{A}
H_E	0.678±0.026	0.638±0.031	0.672±0.019	ns ^{A}
H_O	0.678±0.027	0.635±0.031	0.568±0.030	ns ^{A}
N_A	3.43±0.25	3.42±0.29	3.67±0.19	ns ^{A}
F_{IS}	**-0.009±0.044**	**-0.035±0.020**	**0.171±0.033**	* *^{A}
F_{ST}	0.179±0.018	0.164±0.017	0.151±0.017	ns ^{A}
M	9.20±0.41	10.83±0.82	9.63±0.41	ns $^{K-W}$
m	0.49±0.09	0.52±0.07	0.58±0.06	ns ^{A}

F. sultana

**Parameters**	***Norfolk***	***Cumbria***	***GG***	***P-value***
N_C	**6.80±1.40**	**16.20±1.25**	**8.44±1.62**	***$^{K-W}$
R	**0.22±0.05**	**0.54±0.04**	**0.27±0.07**	*** ^{A}
H_E	0.684±0.017	0.620±0.033	0.698±0.043	ns ^{A}
H_O	**0.655±0.019**	**0.545±0.023**	**0.617±0.040**	* ^{A}
N_A	3.60±0.38	5.25±0.40	4.52±0.72	ns ^{A}
F_{IS}	0.059±0.031	0.121±0.030	0.129±0.077	ns ^{A}
F_{ST}	0.138±0.012	0.158±0.023	0.121±0.013	ns ^{A}
M	14.67±0.74	16.75±1.92	17.69±2.10	ns ^{A}
m	0.62±0.08	0.46±0.07	0.63±0.09	ns ^{A}

C. mucedo

**Parameters**	***DHC***	***HC***	***I***	***P-value***

F. sultana

**Parameters**	***DHC***	***I***	***P-value***

N_C	**6.86±0.84**	**6.57±0.74**	**4.29±0.52**	* ^{A}	N_C	11.11±1.50	8.60±1.50	ns $^{K-W}$
R	**0.22±0.03**	**0.20±0.02**	**0.12±0.02**	* ^{A}	R	0.35±0.05	0.30±0.06	ns ^{A}
H_E	**0.726±0.013**	**0.662±0.016**	**0.614±0.030**	** ^{A}	H_E	0.693±0.014	0.639±0.033	ns ^{A}
H_O	0.628±0.028	0.622±0.020	0.591±0.030	ns ^{A}	H_O	0.623±0.015	0.600±0.033	ns ^{A}
N_A	**4.14±0.23**	**3.51±0.18**	**3.06±0.21**	** ^{A}	N_A	4.85±0.36	3.68±0.46	ns ^{A}
F_{IS}	0.152±0.048	0.059±0.038	0.011±0.056	ns ^{A}	F_{IS}	0.113±0.025	0.077±0.051	ns ^{A}
F_{ST}	**0.146±0.012**	**0.229±0.018**	**0.247±0.020**	*** ^{A}	F_{ST}	**0.166±0.019**	**0.250±0.031**	* ^{A}
M	**11.89±0.91**	**9.31±0.40**	**9.13±0.44**	* $^{K-W}$	M	15.98±0.90	14.96±1.20	ns ^{A}
m	0.66±0.07	0.55±0.06	0.43±0.08	ns ^{A}	m	0.55±0.06	0.62±0.08	ns ^{A}

(b)

C. mucedo					*F. sultana*				
**Parameters**	***Norfolk***	***Cumbria***	***GG***	***P-value***	**Parameters**	***Norfolk***	***Cumbria***	***GG***	***P-value***
N_C	5.75±0.90	6.08±0.94	5.33±0.64	ns ^{A}	N_C	**6.80±1.40**	**16.20±1.25**	**8.44±1.62**	*** $^{K-W}$
R	0.16±0.03	0.18±0.03	0.16±0.02	ns ^{A}	R	**0.22±0.05**	**0.54±0.04**	**0.27±0.07**	*** ^{A}
H_E	0.678±0.026	0.638±0.031	0.688±0.018	ns ^{A}	H_E	0.684±0.017	0.620±0.033	0.698±0.043	ns ^{A}
H_O	0.678±0.027	0.635±0.031	0.598±0.024	ns ^{A}	H_O	**0.655±0.019**	**0.545±0.023**	**0.617±0.040**	* ^{A}
N_A	3.43±0.25	3.42±0.29	3.64±0.20	ns ^{A}	N_A	3.60±0.38	5.25±0.40	4.52±0.72	ns ^{A}
F_{IS}	**-0.009±0.044**	**-0.035±0.020**	**0.149±0.050**	* ^{A}	F_{IS}	0.059±0.031	0.121±0.030	0.129±0.077	ns ^{A}
F_{ST}	0.179±0.018	0.164±0.017	0.119±0.016	ns ^{A}	F_{ST}	0.138±0.012	0.158±0.023	0.121±0.013	ns ^{A}
M	9.20±0.41	10.83±0.82	9.47±0.53	ns $^{K-W}$	M	14.67±0.74	16.75±1.92	17.69±2.10	ns ^{A}
m	0.49±0.09	0.52±0.07	0.64±0.08	ns ^{A}	m	0.62±0.08	0.46±0.07	0.63±0.09	ns ^{A}

C. mucedo

Parameters	*DHC*	*HC*	*I*	*P-value*
**N_C**	6.09±0.77	6.57±0.90	4.75±0.65	ns^{A}
R	0.18±0.03	0.20±0.03	0.13±0.03	ns^{A}
**H_E**	**0.725±0.016**	**0.652±0.018**	**0.532±0.034**	* ^{A}
**H_O**	0.652±0.030	0.638±0.024	0.621±0.024	ns ^{A}
**N_A**	**3.95±0.25**	**3.44±0.17**	**3.14±0.25**	* ^{A}
**F_{IS}**	0.122±0.055	0.014±0.041	(-)0.036±0.064	ns ^{A}
**F_{ST}**	**0.142±0.015**	**0.230±0.019**	**0.249±0.019**	*** ^{A}
M	**12.14±1.05**	**9.40±0.44**	**8.54±0.47**	* $^{K\text{-}W}$
m	0.69±0.09	0.54±0.07	0.48±0.09	ns ^{A}

F. sultana

Parameters	*DHC*	*I*	*P-value*
**N_C**	11.11±1.50	8.60±1.50	ns $^{K\text{-}W}$
R	0.35±0.05	0.30±0.06	ns ^{A}
**H_E**	0.693±0.014	0.639±0.033	ns ^{A}
**H_O**	0.623±0.015	0.600±0.033	ns ^{A}
**N_A**	4.85±0.36	3.68±0.46	ns ^{A}
**F_{IS}**	0.113±0.025	0.077±0.051	ns ^{A}
**F_{ST}**	**0.166±0.019**	**0.250±0.031**	* ^{A}
M	15.98±0.90	14.96±1.20	ns ^{A}
m	0.55±0.06	0.62±0.08	ns ^{A}
