# Supplementary material for: To remain or leave: Dispersal variation and its genetic consequences in benthic freshwater invertebrates
Source: Ecol Evol. 2019 Oct 18;9(21):12069–88. doi: 10.1002/ece3.5656 (PMC6854113; doi:10.1002/ece3.5656)
Supplement: Supplementary file 5 [file ECE3-9-12069-s005.pdf]

Table S5. Test for genetic bottlenecks in a) *Cristatella mucedo* and b) *Fredericella sultana* populations. Wilcoxon (one-tailed and two-tailed) tests under the Two-phase Mutation model (TPM) and the Stepwise Mutation model (SMM). A Mode-shift test for detection of shifted allele-frequencies was also conducted. Red lettering identifies samples providing evidence of recent bottlenecks according to each test. DHC = Directly Hydrologically Connected; HC = Hydrologically Connected; I = Isolated.

(a)	TPM		SMM		Mode-shift
Site name	Wilcoxon P-value one tail	Wilcoxon P-value two tails	Wilcoxon P-value one tail	Wilcoxon P-value two tails	
Norfolk *DHC*					
**CKS**	0.005	0.010	0.005	0.010	Shifted
**SWA**	0.007	0.014	0.042	0.083	Shifted
HOV	0.024	0.049	0.125	0.250	Shifted
WRO	0.053	0.105	0.080	0.160	Shifted
HC					
**UPG**	0.005	0.010	0.042	0.083	Shifted
ORS	0.014	0.027	0.150	0.300	Shifted
**CRO**	0.007	0.014	0.016	0.032	Shifted
BTF	NA	NA	NA	NA	NA
I					
**GPA**	0.002	0.004	0.002	0.004	Shifted
WOL	0.001	0.002	0.053	0.105	Shifted
SEL	NA	NA	NA	NA	NA
BCK	NA	NA	NA	NA	NA
Cumbria *DHC*					
RYD	0.010	0.020	0.064	0.129	Shifted
WIN	0.053	0.105	0.100	0.193	Shifted
**EST**	0.001	0.002	0.001	0.002	Shifted
GSM	NA	NA	NA	NA	NA
HC					
**CON**	0.001	0.002	0.010	0.020	Shifted
**LOU**	0.002	0.004	0.002	0.004	Shifted
**LOL**	0.009	0.019	0.042	0.083	Shifted
**BAS**	0.014	0.027	0.024	0.049	Shifted
BRO	0.016	0.032	0.053	0.105	Shifted
I					
**MOK**	0.003	0.006	0.024	0.048	Shifted
ULP	NA	NA	NA	NA	NA
WHF	NA	NA	NA	NA	NA
HTF	NA	NA	NA	NA	NA
Glasgow					

DHC					
**LND**	0.002	0.004	0.003	0.006	Shifted
WDN	NA	NA	NA	NA	NA
BIS	NA	NA	NA	NA	NA
HC					
**HOG**	0.007	0.014	0.012	0.024	Shifted
**TAN**	0.005	0.001	0.005	0.001	Shifted
**CAR**	0.005	0.010	0.005	0.010	Shifted
GNQ	0.042	0.084	0.042	0.084	Shifted
CSE	NA	NA	NA	NA	NA
I					
BAR	0.003	0.006	0.102	0.203	Shifted
**BAN**	0.001	0.002	0.005	0.010	Shifted
**CRR**	0.005	0.001	0.005	0.001	Shifted
**LIB**	0.005	0.001	0.005	0.001	Shifted
N. Ireland					
DHC					
TIR	0.285	0.570	0.285	0.570	Shifted
CAB	0.246	0.492	0.348	0.695	Shifted
DEJ	NA	NA	NA	NA	NA
HC					
GOL	0.014	0.027	0.101	0.203	Shifted
SAN	0.116	0.232	0.246	0.492	Shifted
**MIL**	0.001	0.002	0.002	0.004	Shifted
DRO	NA	NA	NA	NA	NA
I					
CGN	0.010	0.020	0.064	0.129	Shifted
**LEN**	0.004	0.008	0.008	0.016	Shifted
MOO	NA	NA	NA	NA	NA
BRY	NA	NA	NA	NA	NA

(b)<br>Site name	TPM<br>Wilcoxon P-value one tail	Wilcoxon P-value two tails	SMM<br>Wilcoxon P-value one tail	Wilcoxon P-value two tails	Mode-shift test
Norfolk					
DHC					
**WEN**	0.001	0.001	0.005	0.001	Shifted
WAT	0.005	0.010	0.070	0.131	Shifted
WLY	0.001	0.002	0.042	0.083	L-shaped
BUR	0.003	0.007	0.065	0.131	Shifted
**YAR**	0.002	0.005	0.016	0.032	Shifted
RBW	0.012	0.024	0.116	0.232	Shifted
RBL	NA	NA	NA	NA	NA
SBK	NA	NA	NA	NA	NA
CHT	NA	NA	NA	NA	NA
STI	NA	NA	NA	NA	NA
I					
**GPK**	0.001	0.001	0.001	0.001	Shifted
RTH	0.001	0.002	0.053	0.105	Shifted
**GLA**	0.002	0.003	0.012	0.024	Shifted
NAR	NA	NA	NA	NA	NA
WAV	NA	NA	NA	NA	NA
Cumbria					
DHC					
RCO	0.002	0.005	0.138	0.275	L-shaped
WIM	0.016	0.032	0.461	0.922	L-shaped
BOW	0.012	0.024	0.246	0.492	L-shaped
BRA	0.002	0.005	0.116	0.232	L-shaped
**RDW**	0.001	0.001	0.012	0.024	Shifted
ROT	0.003	0.010	0.122	0.238	L-shaped
I					
**HAR**	0.003	0.007	0.042	0.084	Shifted
**YEW**	0.001	0.001	0.001	0.001	Shifted
**CRK**	0.001	0.001	0.001	0.002	Shifted
**PBK**	0.001	0.001	0.001	0.001	Shifted
Glasgow					
DHC					
**CRN**	0.003	0.007	0.012	0.024	Shifted
RCD	0.012	0.024	0.385	0.770	Shifted
FCC	0.005	0.010	0.138	0.275	Shifted
**FCD**	0.001	0.001	0.003	0.007	L-shaped
I					
**BBB**	0.001	0.002	0.001	0.002	Shifted
UNC	0.007	0.014	0.615	0.846	Shifted
**NCW**	0.002	0.005	0.016	0.032	Shifted
LUW	NA	NA	NA	NA	NA

BLW	NA	NA	NA	NA	NA
