# Supplementary material for: To remain or leave: Dispersal variation and its genetic consequences in benthic freshwater invertebrates
Source: Ecol Evol. 2019 Oct 18;9(21):12069–88. doi: 10.1002/ece3.5656 (PMC6854113; doi:10.1002/ece3.5656)
Supplement: Supplementary file 6 [file ECE3-9-12069-s006.PDF]

Supplemental Information for:

To remain or leave: dispersal variation and its genetic consequences in benthic freshwater invertebrates

Paolo Ruggeri[1, 2], Ellen Pasternak[1,3], Beth Okamura[1]

1 Department of Life Sciences, Natural History Museum, Cromwell Road, SW7 5BD, London, United Kingdom
2 Laboratory of Integrative Biology of Marine Models, CNRS-Sorbonne University, Station Biologique de Roscoff, Place Georges Teissier, 29680, Roscoff (France).
3 Zoology Department, Oxford University, 11a Mansfield Road, Oxford OX1 3SZ

Corresponding Author: Beth Okamura

Table of Contents:

**DNA Extraction protocol**	Page 1
**PCR protocol and electrophoresis for screening PCR samples**	Page 2

DNA Extraction protocol

The DNA extraction for both *Cristatella mucedo* statoblasts and *Fredericella sultana* branches were carried out with a HOT-SHOT method.

Individual statoblasts or branches were placed in individual Eppendorf tubes and rinsed for 10 minutes in 1X TE-buffer (Tris-EDTA Buffer; 1M Tris pH 8.0, 0.5M EDTA pH 8.0). *C. mucedo* statoblasts were then crushed against the tube wall, while *F. sultana* branches were minced and homogenized using mono-use sterile tips. A volume of 100 µl of 1X Lysis-buffer (25mM Sodium hydroxide, 0.2mM disodium-EDTA) was added to each sample and samples were incubated (for the thermal lysis) at 95°C for at least 40 minutes in a thermal shaker.

After the thermal lysis the tubes were spun and an equal volume (100 µl) of neutralizing-buffer (40mM TRIS-HCl) was added to each tube in order to stop the degrading activity performed by sodium hydroxide on the specimen's tissues. DNA precipitation was achieved by adding 500 µl of absolute EtOH previously stored at -20°C and enhanced by maintaining the tubes at -80°C for at least 30 minutes. The tubes were subsequently placed in a refrigerated centrifuge (Eppendorf Centrifuge 5804R) and spun at 8000 rpm for 1 hour at the stable temperature of -7°C.

The supernatant was discarded and the pelleted DNA cleaned twice by adding 100 µl of cool (-20°C) 70% EtOH then centrifuging at 12300 rpm for at least 5 minutes. The EtOH was discarded and samples were left to dry overnight in a sterile incubator at 37°C. The extracted

DNA was then diluted in sterile water. A volume of 50 µl was added to the DNA extracted from C. mucedo statoblasts. The volume of sterile water added to F. sultana DNA varied from 50-150 µl depending on the size of F. sultana branches.

PCR protocol and electrophoresis for screening PCR samples

PCR of samples of C. mucedo and F. sultana was performed using an Applied Biosystems ProFlex PCR System thermal cycler. For both species the markers were multiplexed in two reactions: A1 and B1 contained 3 pairs of primers and A2 and B2 contained 2 pairs of primers (Table S2). PCRs for A1 and B1 were conducted using a final volume of 15 µl, and for A2 and B2 using a final volume of 10 µl. The PCR reaction was conducted using approximately 1 U of GoTaq® Flexi DNA Polymerase (Promega), 0.5 µmol·L–1of each primer, 0.2 mmol·L–1of each dNTP, 1X Colorless GoTaq® Flexi Buffer and 1.5 mmol·L–1 MgCl2. A volume of 3 µl of DNA template was added to multiplexes A1-B1, and a volume of 2 µl of DNA template to multiplexes A2-B2. A blank control was included in each set of PCR reactions.

PCR conditions were optimized for all the microsatellite multiplexes using a touchdown amplification profile (reducing 0.5°C per cycle the initial annealing temperature). The PCR profile involved an initial denaturation step at 95°C for 5 min, followed by 10 cycles of 95°C x 30 seconds of denaturation, 45 seconds of annealing (see Table S2 for starting annealing temperature) and 72°C x 45 seconds of extension. An additional 25 cycles were performed with the annealing temperature fixed at 5°C less than the starting annealing temperature. A final elongation step at 72°C for 5 minutes was performed. PCR products were visualised in a 2% agarose gel stained with GelRed® Nucleic Acid Gel Stain (Biotium) and a 100 bp step ladder marker (HyperLadder™ 100bp, Bioline) for size determination.
